# Behavioral Signatures of Values in Everyday Behavior in Retrospective and Real-Time Self-Reports

**DOI:** 10.3389/fpsyg.2019.00281

**Published:** 2019-02-19

**Authors:** Ewa Skimina, Jan Cieciuch, Shalom H. Schwartz, Eldad Davidov, René Algesheimer

**Affiliations:** ^1^Institute of Psychology, Cardinal Stefan Wyszyński University in Warsaw, Warsaw, Poland; ^2^URPP Social Networks, University of Zurich, Zurich, Switzerland; ^3^Department of Psychology, Hebrew University of Jerusalem, Jerusalem, Israel; ^4^Institute of Sociology and Social Psychology, Faculty of Management, Economics and Social Sciences, University of Cologne, Cologne, Germany; ^5^Department of Sociology, University of Zurich, Zurich, Switzerland

**Keywords:** values, daily behavior, value trait, value state, experience sampling method, act frequency approach, value-behavior relations

## Abstract

We identified behavioral signatures of the values distinguished in the Schwartz et al. refined value theory (2012). We examined behavioral signatures for two types of values, value states and value traits. We conducted two studies using innovative approaches. Study 1 used retrospective self-reports whereas Study 2 used self-reports in real time. In Study 1 (*N* = 703), we sought act frequency signatures of the 19 basic value traits that the Portrait Value Questionnaire-Revised (Schwartz, 2017) measures. We examined the frequency of 209 acts from the Oregon Avocational Interest Scales (Goldberg, 2010) for which there were no expectations that values would necessarily influence them. We computed partial correlations between each behavioral act and each value. We discuss the theoretical links to each value of the 10 behavioral acts that correlated most highly with it. Study 2 analyzed 9,416 behavioral acts of 374 participants. We measured value expressions in current behavior, i.e., *value states*, using experience sampling methodology (ESM). We asked participants 7 times per day for 7 days what they had been doing during the past 15 min and how important 9 different values from the Schwartz's refined value theory were to them during that activity. Because the questions about activities were open-ended, the set of behavioral acts analyzed in Study 2 was theoretically unlimited. To find signatures of values in behavior, we identified the activities during which participants reported the highest level of importance for each value. Both studies revealed meaningful associations between values and daily behavior.

## Introduction

### Approaches to Studying Value-Behavior Relations

Values refer to what people find important in their life (Cieciuch and Schwartz, 2017). The most popular definitions of values link them to motivation. Allport (1961) referred to values as motivational forces that dominate in life. Schwartz (1992) defined them as transsituational life goals that guide human perception and behavior. As motivational constructs, values are expected to influence behavior (Bardi and Schwartz, 2003; Roccas and Sagiv, 2010, 2017; Cieciuch, 2017). The potential of values to influence behavior is one of the features that make them interesting for psychologists.

There are two main approaches to studying value-behavior relations. One seeks to explain particular behaviors with the help of various values. The other seeks to identify behaviors that express a particular value. Both lines of research have yielded interesting results that have expanded our knowledge concerning value-behavior relations.

Most studies adopt the first approach. They examine the relationships between a specific behavior and a set of values in order to find those values, if any, that are useful for explaining the variance in the specific behavior. The main aim of such studies is to explain a particular behavior. For instance, in the context of social interaction, values have been used to explain helping behavior (Daniel et al., 2015), prosocial behavior (Lönnqvist et al., 2013), and antisocial behavior (Molero Jurado et al., 2017). Values have also been used to explain socially undesirable behaviors such as aggression or violence (Benish-Weisman, 2015; Seddig and Davidov, 2018, in this volume), unethical behavior (Feldman et al., 2015), alcohol consumption (Nordfjærn and Brunborg, 2015), and risky sexual behavior (Goodwin et al., 2002). Associations between values and specific behaviors have been studied in a wide variety of life domains. These include sports (Šukys and Majauskienė, 2014), learning (Fries et al., 2005), leisure activities (Rechter and Sverdlik, 2016), internet use (Hartman et al., 2006), consumer participation in virtual communities (Mai and Olsen, 2015), concern for the environment (de Groot and Steg, 2008), voting (Schwartz et al., 2010), parenting (Gaunt, 2005), and health (Bogg et al., 2008), for example. Researchers have also used values to explain attitudes toward behaviors, assuming that those attitudes may indicate likely behavior (e.g., discrimination against minorities, Beierlein et al., 2016; e.g., interpersonal violence, Seddig and Davidov, 2018, in this volume).

The second approach to value-behavior relations seeks to validate a specific value model and applies the act frequency approach (AFA). The AFA was introduced in studies of personality traits by Buss and Craik (1983) and adapted for value studies by Bardi and Schwartz (2003). In the AFA, a set of values distinguished in a model constitutes a starting point. The first step is to choose behaviors expected a priori to express primarily each of the values in the model. Such behaviors are called *value-expressive*. The second step is to measure both value preferences and the frequency with which people perform the value-expressive behaviors. This enables testing of hypotheses concerning the validity of a value model. Adopting this approach, Bardi and Schwartz (2003) found that each of the value-expressive behaviors chosen a priori for the 10 values in the Schwartz (1992) model correlated most positively with the particular value that it was expected to express. Each behavior also correlated positively with the adjacent values in the motivational circle. Some values (stimulation, tradition, and hedonism) revealed stronger associations with behavior than others (benevolence, security, achievement, and conformity).

The refined Schwartz model of values (Schwartz et al., 2012; Schwartz, 2017), which distinguishes 19 values, has also been validated using AFA. Schwartz and Butenko (2014) found that relations among value-expressive behaviors are organized in largely the same motivational circle as relations among the personal values are. Schwartz et al. (2017) demonstrated that value-expressive behaviors are often not the product of a single value. Rather, values on one side of the motivational circle promote them whereas values on the opposite side of the circle inhibit them. Thus, behaviors are a product of value trade-offs.

Both approaches described above demonstrate the existence of links between values and behavior. However, they are not free of limitations. The first approach typically correlates values with one behavior at a time, so comparing behaviors in terms of their associations with the set of values is not possible. Moreover, behaviors are chosen for these analyses based on the expectation that they should be influenced by value preferences but no study has explored relations of values to a comprehensive pool of behaviors. The second approach includes numerous behaviors in the analyses, but the pool of behaviors is sharply limited. First, the pool depends on the set of values included in a model. Second, it includes behaviors expected a priori to express primarily one value. Thus, behaviors that several values may jointly promote are usually not included. Consequently, many everyday behaviors are not included in research on value-behavior relations. By excluding the wide range of everyday behaviors, we may fail to uncover interesting information about value-behavior relations. The current study proposes another approach to studying value-behavior relations, one that can reveal value signatures in everyday behavior.

Multiple values may indeed propel most everyday behaviors (Bardi and Schwartz, 2003). Some behaviors may even express opposing values (Lönnqvist et al., 2013). Lönnqvist et al. (2013) differentiate value-expressive from value-ambivalent behaviors. The latter are behaviors that might be motivated by values on one side of the value circle for some people and by values on the other side of the circle for others. Lönnqvist et al. argue that value-ambivalent behaviors should not correlate significantly with values, because the value associations for some people cancel the opposing associations for other people. Hence, although correlational analysis can uncover the behavioral signatures of value-expressive behaviors, it cannot uncover the signatures of value-ambivalent behaviors. The approach applied in the current study enables identifying both.

This study sought to provide a broader view of the relationships between values and everyday behavior. We conducted exploratory analyses of large pools of behaviors that were not chosen a priori as likely to relate to particular values. The aim was to find the signature of values in everyday behaviors. That is, we sought to uncover the traces of possible value influence on behaviors sampled from everyday experiences, regardless of their presumed relevance to values. For this purpose, we applied the following two approaches: (a) act frequency assessment for a set of behaviors developed to represent the range of everyday experience, and (b) experience sampling in real-time. The first approach was inspired by research on the relations of everyday behavior to personality traits (Chapman and Goldberg, 2017; Elleman et al., 2017). The second drew on the differentiation between value traits and value states introduced by Skimina et al. (2018a).

### Value Traits and Value States

According to Schwartz (Schwartz, 1992, 1996; Schwartz et al., 2012), values are beliefs, representing desirable, abstract goals that motivate behavior by serving as standards according to which people behave and evaluate actions, other people, and events. Values are transsituational. That is, they are relevant across different contexts—for instance, in the workplace or at school, in leisure activities, with friends or strangers. This feature distinguishes values from norms and attitudes, which refer to specific actions or situations.

However, Schwartz describes not only values as abstract goals but also values in action. He avers that “values influence action when they are relevant in the context (hence likely to be activated) and important to the actor” (Schwartz, 2012, p. 4). These two claims—that values are transsituational, abstract goals but are activated only when relevant in a specific context—might seem inconsistent at first sight. The apparent inconsistency disappears, however, if we distinguish value *traits* from value *states*.

The terms *value traits* and *value states* were introduced only recently by Skimina et al. (2018a). However, the Schwartz theory referred implicitly to both constructs from the very beginning, although it did not make this distinction explicit. The popular definition of values as decontextualized life goals that vary in importance as guides to perception and behavior over time and situations refers to value traits—values as general dispositions. Value traits are similar to personality traits—their role can be discerned in patterns of behaviors aggregated over time and occasions. Personality traits may relate differently to behavior at different points in time. Behavior reflects the interaction of personality traits with situational cues. For that reason, it is justifiable to talk about traits as dispositions, but also about personality states, which are short duration expressions of personality traits (Fleeson and Gallagher, 2009).

According to the Trait Activation Theory (Tett and Guterman, 2000), a trait can be expressed in trait-relevant situations. This means that people with a strong tendency to be aggressive do not always behave aggressively, but they are more sensitive to aggression-inducing stimuli. The same is true in the case of personal values. People differ in value traits, namely they ascribe different levels of importance to different values. This is reflected in the general patterns of their behavior aggregated over time. However, if we want to analyze the relationship between values and single behaviors, we need to take into consideration the situational cues that might activate the value trait. For instance, if a person values benevolence highly, it does not mean that this person behaves benevolently on every occasion. Rather, it means he or she is particularly sensitive to benevolence triggers. Thus, a person with a particular value trait may or may not express that trait as a value state, depending on the presence of value relevant cues in a situation. We define value states as goals that vary in importance as guides to single, specific behavioral acts in a real-time context.

We refer to value traits when we are concerned with what is important to a person in general. We refer to value states when we are concerned with what is important to a person at a single time and in a particular context. Value states, like personality states, depend both on traits (dispositions) and on situational cues.

When Schwartz (1992) formulated one of the crucial assumptions in his theory, he implicitly referred to value states. The theory argues that one source of the circular structure of values is that people cannot promote opposing values in the circle simultaneously, in a single act. The theory accepts that people can attribute some importance to opposing value traits. They can express these opposing value traits as value states that promote action, but these actions occur at different times and in different situations.

Introducing the differentiation between value traits and value states provides a new perspective for studying value-behavior relations. When we focus on values as stable dispositions (value traits), we examine relations between the importance people ascribe to various values and the frequency with which they perform behaviors that express those values across situations. When we focus on values as activated in a situation (value states), we examine relations between the importance people currently ascribe to a value in a specific situation and their behavior in that situation. The study of value states concerns the dynamic interaction between the values people experience immediately and their expression in action.

### Value Traits and Everyday Behavior

In the AFA, the frequency of distinct behaviors performed across a given time period (i.e., a month or a year) is assessed via self-reports on a frequency scale (e.g., 1–3, 4–6 times, and so on). Chapman and Goldberg (2017) used this approach to search for the signatures of the Big Five personality traits in a large pool (400) of everyday behaviors. The behaviors were chosen to provide maximum coverage of experiences without regard for potential underlying traits. The researchers identified the strongest behavioral correlates of each of the Big Five dimensions. They found that some correlates were in line with theoretical expectations (e.g., Emotional Stability correlated negatively with consumption of tranquilizing pills). Other correlates did not fit expectations (e.g., Intellect correlated positively with lounging around the house without clothes on).

Elleman et al. (2017) performed similar analyses using 200 everyday behaviors measured by the Oregon Avocational Interest Scales (ORAIS; Goldberg, 2010). They presented the 10 highest zero-order correlates with behaviors for each of the Big Five dimensions. Most were in line with theoretical expectations. The mean correlations of the 10 most strongly associated behaviors ranged from 0.18 for Emotional Stability to 0.36 for Extraversion. Some behaviors related to more than one personality trait (e.g., planning a party correlated positively with Extraversion and Agreeableness).

We adapted Chapman and Goldberg's (2017) and Elleman et al.'s (2017) approach to search for everyday behavioral signatures of personal values. We used the 209 behavioral item ORAIS (Goldberg, 2010). The frequency of behaviors in ORAIS is assessed over a period of 1 year. We inspected the correlations and identified the behaviors most highly correlated with each of the 19 basic values (value traits). We expected to find stronger relations between value traits and the activities that were likely to express those value traits.

### Value States and Everyday Behavior

Skimina et al. (2018a) utilized the experience sampling method (ESM) to measure value states (momentary importance of values) in real-time behavioral acts. In an experience sampling study, participants report their behavior, thoughts, and feelings multiple times per day for multiple days. This enables assessment of the states (value or personality) experienced and expressed in everyday behavior in natural settings, in real time, and across occasions (Hektner et al., 2007).

Implementing ESM for the study of value-behavior relations presents new, promising possibilities. Participants report their current or immediate, recent behavior. This reduces recall bias. If participants respond to an open-ended question, the pool of behaviors available for analyses is potentially unlimited. Boyd et al. (2015) demonstrated that open-ended responses are more accurate than closed-format self-reports. By using an open-ended format, we can obtain more reliable information on what our respondents are actually doing. Data collected this way is also ecologically valid. Moreover, behaviors reported with experience sampling are common, everyday activities that are rarely included in studies of value-behavior relations. These features can provide a new perspective on value-behavior relations, revealing how routine daily action may be linked to value states. We expected each value state to relate meaningfully to some freely reported behaviors.

### The Schwartz Value Model as a Baseline for Value-Behavior Relations

We used the refined Schwartz (Schwartz et al., 2012; Schwartz, 2017) model of personal values as the theoretical background for our research. This is currently the most widely used value model in psychological research (Brosch and Sander, 2016). Studies of value-behavior relations have used the original version (Schwartz, 1992) or the refined version (Schwartz et al., 2012) of the model. These studies demonstrated associations between values and numerous specific behaviors and confirmed the circular pattern of relationships among value-expressive behaviors (Bardi and Schwartz, 2003; Schwartz and Butenko, 2014; Schwartz et al., 2017). According to Schwartz (1994, 2015), values form a circular continuum that reflects the conflict or compatibility among the motivations underlying value preferences. Any real-time behavioral act can promote values that are adjacent in the circle. However, that act cannot simultaneously promote attainment of opposing values in the circle.

The first study of value states (Skimina et al., 2018a) used the refined Schwartz model. The refined model (Schwartz et al., 2012; Schwartz, 2017) distinguishes 19 basic values. Table 1 presents these values together with their definitions and examples of value-expressive behaviors from previous research that studied value-behavior relations with the AFA approach (Schwartz and Butenko, 2014; Schwartz et al., 2017).

**Table 1:** Nineteen values, their definitions, and exemplary value expressive behaviors.

**Value (Cronbach's α in this study)**	**Definition by motivational goal**	**Exemplary value expressive behaviors**
SDT: self-direction-thought (0.59)	Freedom to cultivate one's own ideas and abilities	Learn something just for the joy of learning.
SDA: self-direction-action (0.72)	Freedom to determine one's own actions	Choose to do a task alone rather than with other people.
ST: stimulation (0.69)	Excitement, novelty, and change	Do risky things for the thrill of it.
HE: hedonism (0.72)	Pleasure and sensuous gratification	Indulge myself by buying things that I didn't really need.
AC: achievement (0.78)	Success according to social standards	Try to impress my boss by working extra hard.
POD: power-dominance (0.83)	Power through control over people	Manipulate others to get what I want.
POR: power-resources (0.82)	Power through control of material and social resources	Buy luxury brands of clothing so that other people will notice.
FAC: face (0.69)	Maintaining one's public image and avoiding humiliation	Wonder whether people are gossiping about me.
SEP: security-personal (0.52)	Safety in one's immediate environment	Avoid walking alone on a dark street at night.
SES: security-societal (0.79)	Safety and stability in the wider society	Express concern about the threat of terrorism or war.
TR: tradition (0.84)	Maintaining and preserving cultural, family, or religious traditions	Celebrated ethnic or religious holidays.
COR: conformity-rules (0.83)	Compliance with rules, laws, and formal obligations	Pay the full entry fee or fare, even when I could get away with not paying it.
COI: conformity-interpersonal (0.79)	Avoidance of upsetting or harming other people	Avoid arguments so that others won't be angry with me.
HU: humility (0.55)	Recognizing one's insignificance in the larger scheme of things	Play down my achievements or talent.
UNN: universalism-nature (0.86)	Preserving the natural environment	Avoid buying items that might harm the environment.
UNC: universalism-concern (0.75)	Equality, justice, and protection for all people	Collect food, clothing, or other things for needy families.
UNT: universalism-tolerance (0.74)	Accepting and understanding those who are different from oneself	Do my best to understand the views of a person with whom I disagree strongly.
BED: benevolence-dependability (0.78)	Being a reliable and trustworthy in-group member	Kept promises I made to friends or family.
BEC benevolence-caring (0.76)	Devotion to the welfare of in-group members	Take care of a friend or family member who was sick.

*Adapted from Schwartz et al. (2017) and Schwartz and Butenko (2014)*.

### Objectives

Past research has sought evidence of value associations with behaviors expected to be influenced by values. The studies presented in this paper applied an exploratory design. Our aim was to find possible signatures of values in everyday behaviors that people experience in their natural settings. We sought to uncover possible relations of values to a sample of everyday behaviors regardless of their relevance to values. For that purpose, we examined sets of naturally occurring behavioral acts. Study 1 investigated acts that were taken from an existing, large inventory of behaviors that was designed to capture the range of major activities in which people engage on a daily basis. We related people's value traits (i.e., chronic value preferences) to these behaviors. Study 2 investigated acts sampled from people's real-time reports of what they were doing in the last few minutes. We related people's value states (i.e., motivating values in the specific situation) to these behaviors. In both studies, we sought to identify (a) those day-to-day behaviors that have value signatures (value traits and value states) and (b) the single or multiple values whose signatures imply that they promote these behaviors or inhibit them.

## Study 1

In Study 1 we used retrospective self-reports of the frequencies of everyday activities and a questionnaire measure of value traits. We sought associations between chronic value preferences and everyday behaviors. We formulated no specific hypothesis regarding possible associations.

### Participants

Participants were 767 Polish individuals age 16 to 72 (*M* = 29.72, *SD* = 12.64), 55.8% female, 42.8% male, and 1.4% providing no gender information. Excluding observations with missing data reduced the sample for the analysis to 703. Participants came from large cities (>100,000 inhabitants, 42%), medium-sized cities (50,000–100,000; 18%), small towns (<50,000; 19%), and villages (21%). Completed education was university (32%), high school (51%), and less than high school (17%). Thirty-eight percent of the sample were single, 28% in an informal relationship, 30% married, 3% divorced, and 1% widowed. Thirty percent of the sample had children and 23% lived with their children. Eighty-nine percent of the sample stated that they believed in God and 97% of them were Roman Catholic.

Trained research assistants recruited the participants. The assistants were psychology students who participated in the study in exchange for course credits. Each student administered paper-and-pencil questionnaires to ~6–10 persons chosen from a pool of their distant relatives, friends, and acquaintances. Participants signed nicknames on the questionnaire, so researchers had no access to personal data, thereby insuring anonymity. Participation in the study was entirely voluntary.

The study complied with the recommendations of the Commission of Ethics and Bioethics at the Cardinal Stefan Wyszyński University in Warsaw. The institutional guidelines for research on adults did not require formal approval by the Commission for this study. All participants provided their oral consent.

### Measures

#### Value Traits

We used the Polish version (Cieciuch, 2013) of the Portrait Values Questionnaire—Revised (PVQ-RR; Schwartz, 2017) to measure the 19 values in the refined version of Schwartz's theory. The questionnaire consists of 57 items describing different people according to what is important to them. Respondents indicated how similar the person described in an item was to themselves, using a 6-point scale (from 1—*not like me at all* to 6—*very much like me*). Cronbach alpha coefficients for each of the 19 three-item value scales ranged from 0.52 (for security-personal) to 0.86 (for universalism-nature) with a mean of 0.74. Table 1 lists all alpha coefficients.

#### Behavioral Acts

To measure the frequency of behavioral acts, we utilized the 209 items from the Oregon Avocational Interest Scales (ORAIS; Goldberg, 2010), adapted to the Polish culture by Skimina et al. (2017). Each item describes a behavioral act that can be performed on a daily basis. The participants were asked to assess how frequently they performed each behavior, using the following scale: 1 – *never in my life*, 2 – *not in the past year*, 3 – one *or two times in the past year*, 4 – three *to ten times in the past year*, 5 – *more than ten times in the past year*. Goldberg grouped the items into 33 scales (e.g., creative activity, social networking, or summer activities), but we used the 209 items as indicators of single behavioral acts and conducted more detailed analyses on the single items separately.

### Procedure

Participants filled out a set of questionnaires in three sessions, each separated by ~2 weeks. During the first session, they completed a personality inventory not relevant to this study. During the second session they completed the ORAIS questionnaire. During the third session, they first completed a personality inventory not relevant to this study and then the PVQ-RR. Completing the questionnaires in different sessions reduced the likelihood of consistency and shared bias. Measuring the frequency of behavior 2 weeks prior to measuring value preferences should not affect the results, because values as dispositions (i.e., value traits) are relatively stable over time and the retrospective behavior measure covered a period of 1 year.

### Analyses

We analyzed relations of value traits to frequencies of behavioral acts by computing partial correlations of each value with each behavioral act, controlling for age and gender. This follows the approach of Chapman and Goldberg (2017) who controlled for a set of demographics when analyzing the relations of personality traits to frequencies of behavioral acts.

We centered value scores within-persons to control for individual response tendencies and to take into account the interrelatedness of the values within the circular structure. We did this by subtracting the mean rating that each respondent gave to all values from that respondent's rating of each value. Centering is a common practice applied in analyses of circular models of personality traits and of values (Alden et al., 1990; Schwartz, 1992; Moskowitz, 1994; DeYoung et al., 2013; Strus and Cieciuch, 2017). Centering usually shrinks expected positive associations and increases expected negative associations (He et al., 2017). These modifications typically reflect the expected tradeoff of opposing values. As such, centering is desirable when relating values to other variables. The Supplementary Material presents the results of an analysis conducted on raw value scores (Table B).

### Results and Discussion

For each of the 19 value traits, we selected the 10 behavioral acts with which it correlated most highly. Several behavioral acts appeared among the 10 highest correlates for more than one value trait. A total of 89 distinct behavioral acts constituted the set of acts that made up the 10 highest correlates of all the value traits. Table 2 presents a partial correlation matrix (controlling for age and gender) of the 89 selected behavioral acts with the 19 (centered) value traits. Both partial and zero-order correlation coefficients are reported in Table A in the Supplementary Material. The 10 highest correlates of each value are shown in bold. At the bottom of the tables, we present correlation coefficients of each value trait with the aggregate of its 10 most highly correlated behaviors. In the tables, we grouped behavioral acts with related content.

**Table 2:** Partial correlations (controlling for age and gender) between centered value traits and 89 behavioral acts.

**No**	**Item**	**SDT**	**SDA**	**ST**	**HE**	**AC**	**POD**	**POR**	**FAC**	**SEP**	**SES**	**TR**	**COR**	**COI**	**HU**	**UNN**	**UNC**	**UNT**	**BEC**	**BED**
**CHILDREN**																				
175	Read the comics to a child.	0.06	−0.05	−0.08	−0.09	**−0.16**	−0.05	−0.07	−0.04	0.02	0.01	0.11	0.08	0.01	0.04	0.01	0.09	0.09	0.02	0.04
**HOMEMAKING**																				
5	Washed dishes.	0.06	−0.02	−0.10	−0.14	−0.07	**−0.14**	−0.13	−0.04	−0.02	0.08	0.11	0.05	0.04	0.04	0.01	**0.14**	**0.13**	0.04	0.09
9	Cared for a potted plant.	0.09	−0.05	−0.06	−0.06	−0.09	**−0.15**	−0.12	−0.06	−0.03	0.03	0.01	0.05	0.07	0.01	**0.16**	0.08	0.08	0.10	0.05
38	Made a bed.	0.02	−0.04	−0.04	−0.07	−0.01	**−0.17**	−0.12	0.03	−0.03	0.03	0.07	0.04	0.06	0.05	0.01	0.10	**0.13**	0.07	0.02
70	Cleaned the house.	0.09	−0.04	0.00	−0.08	−0.08	**−0.16**	−0.13	−0.09	−0.02	0.02	0.09	0.01	0.02	0.06	0.05	0.09	**0.16**	0.08	0.06
103	Ironed linens or clothes.	0.05	−0.10	−0.08	−0.15	−0.04	**−0.17**	−0.10	−0.10	−0.03	0.11	**0.20**	0.09	0.05	0.05	0.01	0.12	0.09	0.05	0.08
135	Cooked a meal.	0.07	0.01	−0.09	−0.09	−0.06	**−0.13**	−0.14	−0.05	−0.02	0.07	0.11	0.09	0.00	0.05	0.05	0.08	0.09	0.04	0.06
167	Baked a cake, pie, cookies, or bread.	0.01	−0.09	−0.04	−0.08	−0.03	−0.10	−0.05	**−0.12**	−0.04	0.04	0.08	0.07	0.05	0.04	0.07	0.04	0.05	0.06	0.05
**GARDENING**																				
42	Gardened.	−0.03	−0.10	0.01	−0.06	−0.04	−0.04	−0.06	−0.08	−0.03	0.02	0.10	0.06	−0.01	0.03	**0.17**	0.01	0.00	0.04	−0.02
74	Did yard work.	−0.06	**−0.13**	0.00	−0.05	−0.08	−0.02	−0.05	−0.07	0.01	−0.02	0.09	0.07	0.03	0.05	0.12	0.03	0.00	0.06	0.00
107	Planted or transplanted a plant.	−0.02	−0.10	−0.06	−0.13	−0.07	−0.06	−0.06	−0.04	0.04	0.01	0.07	0.05	0.08	0.05	**0.16**	0.03	0.01	0.03	−0.01
171	Bought plants for a garden or yard.	0.02	**−0.12**	0.03	−0.04	−0.04	0.03	0.00	−0.11	0.03	−0.08	0.07	0.04	0.04	−0.01	0.09	−0.03	0.00	0.01	−0.02
**SHOPPING**																				
122	Ordered food to be delivered.	0.03	0.01	0.08	0.12	0.09	−0.01	0.07	−0.03	−0.07	−0.07	−0.01	−0.03	−0.03	**−0.14**	−0.02	−0.09	0.06	0.01	0.04
130	Purchased a musical album.	0.16	0.06	0.10	−0.01	−0.04	0.04	−0.06	−0.07	**−0.14**	−0.08	0.01	0.02	−0.03	−0.12	0.00	0.00	**0.13**	0.01	−0.01
162	Shopped in a music store.	0.18	0.02	0.12	0.02	−0.04	−0.01	−0.07	**−0.13**	−0.12	−0.06	−0.02	−0.03	−0.03	−0.06	0.05	0.02	**0.12**	0.05	0.02
179	Shopped on the web.	0.16	0.08	0.03	−0.02	0.11	0.00	0.06	−0.03	−0.08	0.01	0.00	−0.02	**−0.15**	−0.13	−0.02	−0.03	0.04	0.02	0.01
189	Examined the clothing categories on eBay.	0.03	0.02	0.12	0.08	**0.16**	−0.02	0.12	0.09	−0.04	0.01	−0.01	−0.10	−0.11	**−0.14**	−0.03	−0.09	−0.01	−0.05	−0.08
206	Used eBay to buy or sell something.	0.13	0.08	0.06	0.03	**0.15**	0.00	0.08	−0.01	−0.07	−0.03	−0.03	−0.05	**−0.15**	−0.13	−0.01	−0.06	0.03	−0.02	0.02
**BEING ALONE**																				
49	Went to the movies alone.	0.11	0.05	0.08	0.00	−0.06	0.02	−0.05	**−0.15**	0.00	−0.05	−0.06	−0.02	0.01	0.02	0.01	−0.01	0.09	−0.02	0.04
88	Went to a concert or theater alone.	0.20	−0.02	0.12	−0.04	−0.14	0.03	−0.07	**−0.12**	−0.10	−0.05	0.00	−0.01	0.03	0.00	0.03	0.01	0.09	−0.02	−0.02
**DRINKING ALCOHOL**																				
62	Drank whiskey, vodka, gin, or other hard liquor.	−0.03	0.02	0.12	**0.18**	0.15	0.09	**0.19**	0.05	−0.02	−0.10	−0.05	**−0.15**	**−0.13**	−0.11	−0.08	**−0.12**	−0.03	−0.02	−0.06
92	Drank in a bar or night club.	0.04	0.07	**0.17**	**0.20**	0.08	0.12	0.16	−0.02	−0.05	−0.07	−0.09	**−0.15**	**−0.16**	**−0.14**	−0.05	−0.11	0.00	−0.04	−0.05
125	Became intoxicated.	0.04	0.08	**0.21**	**0.26**	0.13	**0.14**	**0.19**	0.00	−0.08	−0.11	−0.11	**−0.19**	**−0.17**	**−0.15**	−0.04	**−0.16**	−0.02	−0.05	**−0.14**
157	Had a hangover.	−0.04	0.05	**0.18**	**0.22**	0.12	**0.13**	0.17	0.03	−0.04	−0.11	−0.08	−0.12	**−0.14**	−0.12	0.00	−0.12	−0.05	**−0.11**	**−0.16**
187	Drank alcohol during working hours.	0.04	0.01	0.11	0.10	0.01	0.04	0.07	0.03	0.01	0.01	−0.09	**−0.15**	−0.06	−0.05	0.00	−0.02	0.01	−0.04	−0.07
**PHYSICAL ACTIVITY**																				
6	Went running or jogging.	0.07	0.01	0.13	0.03	0.04	0.03	0.05	−0.08	**−0.14**	−0.02	0.10	0.00	−0.09	−0.07	0.07	−0.08	−0.05	−0.09	−0.05
173	Went boating or rafting.	0.21	0.05	0.12	0.04	0.03	0.03	−0.03	0.00	**−0.14**	−0.01	−0.04	−0.06	−0.07	−0.09	−0.02	−0.05	0.04	0.00	0.01
196	Participated in an exercise program.	0.09	0.05	0.14	0.02	0.04	0.02	−0.01	−0.03	−0.08	−0.03	0.05	−0.01	**−0.16**	−0.04	0.08	−0.06	0.01	−0.10	−0.04
**SELF-DEVELOPMENT AND LEARNING**																				
7	Attended a public lecture.	**0.24**	−0.03	0.08	−0.11	0.03	0.00	−0.02	−0.10	**−0.14**	0.05	0.11	−0.03	0.01	−0.07	0.03	0.01	0.00	−0.07	−0.07
40	Visited an art exhibition.	**0.28**	0.07	0.06	0.00	0.00	0.03	−0.06	−0.07	**−0.14**	−0.03	−0.06	−0.06	−0.07	−0.10	0.10	−0.05	0.08	0.02	0.02
72	Visited a museum.	0.22	0.03	0.00	−0.08	−0.07	−0.04	−0.12	−0.01	−0.12	0.04	0.05	0.01	−0.02	−0.08	0.12	0.02	**0.12**	0.01	−0.01
101	Looked something up in an encyclopedia.	0.12	0.00	0.00	−0.11	−0.01	−0.04	−0.13	0.04	−0.10	**0.15**	0.10	0.05	−0.01	−0.04	−0.01	0.06	0.03	−0.01	0.02
113	Read a book.	**0.30**	0.09	0.02	−0.05	−0.01	−0.05	−0.11	−0.04	−0.05	0.04	0.05	−0.03	−0.09	−0.09	0.01	0.00	0.02	0.06	0.08
128	Studied some subject.	**0.29**	0.07	−0.01	−0.07	0.05	−0.06	−0.08	0.00	−0.08	0.08	0.04	0.00	−0.11	−0.11	−0.01	0.05	0.05	0.04	−0.01
145	Read poetry.	**0.23**	0.08	−0.01	−0.10	−0.07	−0.05	**−0.20**	−0.08	**−0.20**	0.06	0.02	0.04	−0.05	−0.05	**0.19**	0.08	0.11	0.06	0.04
160	Learned a new skill.	**0.25**	0.05	0.09	−0.02	0.09	0.00	−0.04	−0.03	−0.08	−0.01	−0.05	−0.04	−0.05	−0.07	−0.01	0.01	0.02	0.01	−0.07
176	Bought a book.	**0.32**	0.06	−0.04	−0.11	−0.06	−0.04	−0.11	−0.08	−0.03	0.05	0.05	0.01	−0.08	−0.09	0.03	0.04	**0.14**	0.02	0.06
197	Read a book about some artistic topic.	**0.29**	0.06	0.13	−0.04	0.01	0.00	−0.09	−0.10	−0.09	0.00	−0.02	−0.06	−0.09	−0.12	0.11	−0.04	0.09	−0.01	−0.01
**CREATIVE HOBBY**																				
50	Produced a work of art.	0.20	0.06	0.06	−0.03	0.06	0.02	−0.08	−0.05	−0.04	0.01	−0.05	−0.04	−0.08	−0.10	**0.13**	−0.04	−0.03	0.04	−0.02
82	Wrote poetry.	**0.24**	0.08	0.04	−0.04	−0.03	0.00	−0.12	−0.08	**−0.14**	−0.03	−0.04	−0.04	−0.07	−0.06	**0.14**	0.02	0.03	**0.12**	0.06
178	Played a musical instrument.	**0.24**	0.04	0.08	−0.08	−0.01	−0.03	−0.14	−0.08	−0.12	0.02	0.04	−0.02	−0.01	−0.10	0.05	0.01	0.06	0.07	0.05
200	Sang or played an instrument in public.	0.16	0.03	0.02	−0.06	−0.08	0.01	**−0.18**	−0.11	**−0.17**	0.03	0.11	0.03	−0.02	0.00	0.02	0.03	0.08	0.09	0.08
**PLAYING GAMES**																				
54	Worked on a jigsaw puzzle.	−0.03	−0.08	−0.03	−0.05	−0.13	−0.02	−0.05	−0.02	0.04	0.06	0.10	**0.14**	0.04	0.02	0.05	0.03	0.00	−0.03	−0.06
69	Gambled with cards or dice.	−0.02	−0.05	0.11	0.09	0.03	0.07	0.08	0.00	−0.04	−0.04	−0.03	**−0.16**	−0.01	0.00	0.00	−0.09	0.00	0.00	−0.07
134	Gambled on a slot machine or video poker game.	−0.05	−0.07	0.15	0.14	0.10	0.10	0.13	−0.01	−0.06	−0.11	−0.08	−0.10	0.03	−0.10	−0.01	**−0.13**	0.03	−0.05	−0.06
166	Went to a casino.	−0.04	−0.06	0.10	0.10	0.02	0.12	0.13	0.01	0.00	−0.07	−0.06	−0.07	0.03	−0.06	0.00	−0.05	−0.05	**−0.12**	**−0.11**
198	Bet money on a sports event.	−0.03	−0.01	**0.17**	0.11	0.03	0.08	0.08	−0.05	0.00	−0.06	−0.02	−0.06	−0.04	−0.03	−0.04	−0.11	−0.01	−0.06	−0.08
**HOBBY**																				
127	Read a fashion-related book.	−0.04	**−0.13**	0.12	0.04	0.06	0.02	0.09	−0.02	0.02	−0.11	0.02	0.01	0.09	−0.05	0.05	−0.05	−0.01	**−0.15**	**−0.15**
194	Went fishing or hunting.	0.00	−0.06	0.03	−0.02	−0.06	0.00	−0.04	−0.05	−0.03	0.00	0.02	0.02	−0.01	0.02	**0.14**	0.02	−0.01	−0.03	−0.02
**MUSIC**																				
207	Used an mp3 player or Ipod.	0.21	**0.14**	0.15	0.12	0.08	0.07	0.02	−0.03	−0.14	−0.08	−0.06	−0.07	**−0.15**	**−0.20**	−0.07	−0.07	0.11	−0.02	0.01
**USING INTERNET (BESIDES COMMUNICATION)**																				
60	Surfed the Internet.	0.07	0.07	−0.06	−0.03	0.06	−0.09	−0.03	0.07	−0.03	0.02	0.00	−0.01	−0.05	−0.03	−0.03	0.00	0.07	**0.10**	0.05
65	Downloaded music from the Internet.	0.08	0.04	**0.18**	0.13	**0.16**	0.04	0.08	−0.01	−0.13	−0.12	−0.07	−0.13	−0.09	−0.08	−0.12	−0.06	0.04	0.02	0.02
75	Made an entry on a personal web-page.	0.09	0.01	0.09	0.01	0.07	0.05	0.04	0.00	−0.06	−0.05	−0.02	0.00	−0.08	**−0.15**	0.00	−0.07	0.04	−0.01	0.01
**SOCIAL INTERACTION**																				
4	Discussed sports.	0.05	0.03	0.03	0.02	−0.04	−0.08	−0.03	**−0.11**	−0.04	0.07	0.08	0.01	−0.06	−0.03	0.05	0.00	0.03	0.04	0.02
10	Used a computer for social networking.	0.00	−0.04	0.14	0.13	0.02	0.10	0.12	−0.02	−0.02	−0.12	−0.10	−0.06	0.00	−0.10	0.00	−0.02	0.06	**−0.12**	**−0.14**
97	Traded music with a friend.	0.06	0.04	**0.19**	0.17	0.10	0.06	0.05	−0.08	**−0.16**	−0.13	−0.07	**−0.13**	−0.05	−0.13	−0.04	−0.06	0.10	−0.01	0.02
108	Participated in an online discussion group.	0.13	−0.02	0.16	0.07	0.06	0.08	0.02	−0.05	−0.11	−0.02	−0.02	−0.08	−0.09	**−0.15**	−0.05	−0.04	0.11	−0.03	−0.01
**DATING AND PARTYING**																				
53	Went on a date.	0.04	0.05	0.09	0.06	0.07	0.07	0.08	0.03	0.01	−0.06	−0.02	−0.03	−0.07	**−0.15**	−0.09	−0.06	−0.01	−0.04	−0.03
78	Went to a large party.	0.02	0.07	**0.17**	0.17	0.12	0.09	0.15	−0.03	−0.09	−0.08	−0.02	**−0.16**	−0.13	**−0.14**	−0.08	−0.11	0.00	−0.04	−0.02
202	Had a blind date.	0.01	0.01	0.09	0.11	−0.01	0.04	0.08	0.02	−0.07	**−0.14**	−0.08	−0.10	0.01	−0.04	−0.04	0.01	**0.12**	−0.04	−0.03
**TRANSPORTATION**																				
22	Used public transportation.	0.11	0.01	−0.02	−0.03	−0.07	−0.09	−0.11	0.00	−0.01	0.04	0.02	−0.06	−0.03	0.01	0.05	0.07	0.07	**0.11**	0.12
59	Rode a motorcycle.	−0.08	−0.03	0.12	0.03	0.07	0.10	0.11	0.03	−0.06	−0.06	0.00	−0.02	−0.04	−0.05	0.02	−0.06	−0.08	−0.04	**−0.12**
138	Traveled by train or plane.	0.17	0.01	0.03	−0.07	−0.01	0.00	−0.06	**−0.12**	−0.08	0.00	0.05	0.02	−0.04	−0.05	0.03	−0.01	0.07	0.02	0.08
**RELIGIOUS PRACTICES**																				
2	Discussed religion or spirituality.	0.12	−0.03	−0.13	−0.16	**−0.17**	−0.11	−0.17	**−0.13**	−0.02	**0.17**	**0.19**	0.09	0.05	0.02	0.10	0.12	0.03	0.05	0.08
33	Prayed (not including blessings at meals).	−0.05	**−0.14**	**−0.16**	**−0.22**	−0.09	−0.10	−0.13	−0.08	0.04	**0.14**	**0.34**	**0.13**	0.13	0.08	0.02	**0.13**	−0.03	0.01	0.03
66	Read the Bible or other sacred text.	0.10	−0.05	**−0.16**	**−0.25**	**−0.17**	−0.10	**−0.28**	−0.09	−0.07	**0.17**	**0.32**	0.10	0.10	0.07	0.07	**0.18**	0.01	0.09	0.10
98	Gave a blessing at a meal.	0.04	**−0.17**	−0.14	**−0.20**	**−0.16**	−0.10	**−0.23**	**−0.14**	−0.09	**0.14**	**0.36**	0.13	**0.18**	0.11	0.04	**0.18**	−0.01	0.07	0.08
131	Attended a church or religious service.	−0.04	**−0.12**	−0.13	**−0.20**	−0.08	−0.08	−0.16	−0.05	−0.03	**0.15**	**0.33**	0.11	0.08	0.10	−0.04	**0.12**	−0.03	0.06	0.08
163	Listened to a religious program on the radio or TV.	0.07	**−0.17**	−0.13	**−0.25**	**−0.21**	−0.04	**−0.23**	**−0.13**	−0.10	**0.19**	**0.37**	0.10	**0.16**	**0.15**	0.08	**0.13**	0.01	−0.03	0.01
193	Read a book about religion or spirituality.	0.18	0.01	−0.12	**−0.24**	**−0.15**	−0.01	**−0.22**	**−0.17**	−0.08	**0.14**	**0.27**	0.05	0.07	0.04	0.08	0.11	0.03	0.00	0.04
**SUPPORTING OTHERS**																				
83	Donated money to charity.	0.06	−0.07	−0.07	−0.12	−0.10	−0.02	−0.06	−0.05	−0.06	**0.13**	0.13	0.07	−0.02	0.02	0.10	0.05	−0.01	−0.01	−0.01
144	Volunteered for a club or organization.	0.09	−0.10	−0.01	−0.13	−0.08	0.01	−0.10	−0.10	**−0.14**	−0.01	0.08	0.03	0.05	0.04	0.11	0.07	0.09	0.04	0.01
**ENVIRONMENTAL ACTIVITIES**																				
55	Composted food scraps or yard waste.	0.10	−0.03	−0.07	−0.12	**−0.15**	−0.11	−0.17	−0.07	−0.03	0.04	0.06	0.10	0.05	0.06	**0.21**	0.00	0.11	0.07	0.02
120	Changed a habit to have less impact on the environment.	0.06	−0.10	−0.01	−0.08	−0.08	−0.05	−0.09	−0.10	0.04	0.07	0.07	0.06	−0.03	−0.01	**0.25**	0.04	0.04	−0.05	−0.07
152	Used both sides of a piece of paper before discarding it.	0.13	−0.01	−0.10	−0.16	−0.04	−0.11	**−0.18**	0.01	0.00	0.13	0.07	0.06	−0.02	0.00	0.10	0.07	0.08	0.09	0.03
192	Picked up litter.	0.06	−0.05	−0.01	−0.08	−0.09	−0.08	**−0.18**	−0.09	−0.05	0.06	0.07	0.06	0.04	0.02	0.10	**0.13**	**0.12**	0.05	0.01
**FINANCE/INVESTMENT**																				
16	Obtained stock market prices.	0.09	−0.03	0.12	0.03	0.00	**0.13**	0.06	0.00	0.01	−0.07	−0.07	−0.03	−0.08	0.00	−0.01	−0.06	−0.01	**−0.10**	−0.10
56	Read a book on a financial topic.	0.11	−0.02	0.08	−0.05	0.04	0.08	0.05	−0.05	−0.02	−0.06	0.03	0.05	−0.07	−0.04	0.03	−0.09	0.01	−0.10	**−0.14**
81	Bought or sold stocks or bonds.	0.09	−0.07	0.05	−0.05	0.00	0.12	0.05	0.03	0.03	−0.01	−0.03	−0.02	−0.06	−0.01	0.03	−0.06	0.00	−0.10	**−0.15**
116	Donated money to a political campaign or cause.	0.02	**−0.16**	0.10	−0.04	−0.03	0.11	0.09	0.03	0.01	−0.05	0.03	−0.04	0.00	0.00	0.05	−0.02	−0.04	**−0.10**	**−0.15**
146	Purchased a commodity as an investment.	0.09	−0.03	0.04	−0.04	0.01	0.06	0.04	−0.01	0.04	0.02	0.01	−0.03	−0.06	−0.04	0.07	−0.07	−0.01	−0.06	**−0.15**
**OTHER**																				
12	Made an entry in a diary or journal.	0.22	0.01	0.02	−0.04	0.00	−0.02	−0.06	−0.03	−0.06	0.01	−0.04	−0.01	−0.05	−0.05	0.01	0.06	**0.12**	0.00	−0.02
17	Tried something completely new.	0.16	0.05	**0.17**	0.09	0.07	0.05	−0.01	0.01	−0.09	−0.05	−0.11	−0.07	−0.10	−0.08	0.03	−0.07	0.02	−0.03	−0.06
95	Bought a self-help book.	0.01	**−0.11**	0.08	0.05	0.05	0.02	0.08	0.01	0.00	−0.04	0.03	0.03	0.01	−0.01	0.01	−0.08	−0.06	**−0.10**	−0.12
110	Wrote a thank-you note.	0.15	−0.09	0.00	−0.08	−0.08	−0.01	−0.13	−0.11	−0.05	0.00	**0.15**	0.03	0.03	0.04	0.05	0.03	0.07	0.00	0.02
174	Worked on a scrap book.	0.06	−0.04	0.01	−0.01	−0.07	−0.02	−0.07	0.00	−0.05	−0.05	**0.18**	0.03	0.04	−0.05	−0.03	−0.02	0.03	0.01	0.06
177	Worked on a retirement plan.	0.04	−0.05	−0.04	−0.09	−0.03	0.08	0.00	−0.02	0.07	−0.01	0.04	**0.14**	−0.06	0.04	0.02	−0.08	−0.06	−0.05	−0.04
180	Participated in an animal show.	−0.01	−0.09	0.05	−0.02	−0.02	0.03	0.00	−0.05	0.01	−0.01	0.03	−0.02	0.01	0.02	**0.13**	−0.02	−0.02	−0.08	−0.08
188	Attended a town meeting.	0.02	−0.09	0.02	0.00	0.00	**0.13**	0.03	−0.05	−0.01	−0.01	0.04	−0.03	−0.02	−0.03	0.02	−0.03	0.02	−0.08	−0.09
Aggregated ten highest behavioral correlates		0.45	0.29	0.33	0.37	0.34	0.32	0.37	0.25	0.28	0.27	0.45	0.30	0.30	0.30	0.34	0.27	0.25	0.31	0.32

*SDT, self-direction-thought; SDA, self-direction-action; ST, stimulation; HE, hedonism; AC, achievement; POD, power-dominance; POR, power-resources; FAC, face; SEP, security-personal; SES, security-societal; TR, tradition; COR, conformity-rules; COI, conformity-interpersonal; HU, humility; UNN, universalism-nature; UNC, universalism-concern; UNT, universalism-tolerance; BED, benevolence-dependability; BEC, benevolence-caring*.

*The 10 highest correlates for each value are in bold. To calculate aggregate correlations we reversed the highest correlates with a negative sign*.

*N = 703. All coefficients > |0.07| are significant at p < 0.05. All coefficients > |0.10| are significant at p < 0.01. All coefficients > |0.12| are significant at p < 0.001*.

As might be expected for a set of behaviors not chosen to be value-expressive, most correlations with values were quite weak. Only tradition and self-direction-thought correlated moderately with a number of behaviors (>|0.30|). The zero-order correlations were higher than the partial correlations for many values (e.g., stimulation, hedonism, achievement, and security-personal). Correlations with aggregated behavioral acts (after reversing the signs of negative correlations) were noticeably higher than correlations with single behavioral acts. Partial correlations ranged 0.25 to 0.45 with a mean of 0.32. Zero-order correlations ranged from 0.23 to 0.47 with a mean of 0.37. This is consistent with results of previous studies in which correlations of attitude, value, or personality variables with behavior were greater for aggregated behaviors (e.g., Weigel and Newman, 1976; Digman, 1990; Skimina et al., 2018b).

Table 2 reveals that the correlations of several values with behaviors were primarily positive (e.g., self-direction-thought, tradition, universalism-nature, and universalism-tolerance). In contrast, the correlations of several other values were primarily negative (e.g., self-direction-action, face, power-resources, conformity-interpersonal, security-personal, and benevolence-dependability). Many behaviors correlated with multiple values, often values that are adjacent in the value circle (e.g., housekeeping behaviors negatively with both types of power values). The behaviors most strongly related to multiple values were religious practices (e.g., praying, reading the Bible) and alcohol consumption (e.g., becoming intoxicated, having a hangover). Notably, both behaviors correlated most positively with a value from one side of the value circle and most negatively with a value from the opposing side. For religious practices it was tradition vs. hedonism, for drinking alcohol it was hedonism vs. conformity-rules.

Additionally, Table B presents the results of a correlational analysis conducted on raw value scores. Eighty-nine distinct behavioral acts constituted the set of acts that made up the 10 highest correlates of the raw value traits. Of these, 72% were also among those for the centered value scores that were listed in Table 2. As expected, there were fewer negative and more positive correlations with the raw (i.e., uncentered) scores. Nonetheless, security-personal, face, and power-resources values had primarily negative correlations with behavioral acts. Below we present and discuss the results based on the centered scores (as commonly done in the research tradition of value-behavior relations).

#### Openness to Change Values

The behavioral acts with the strongest signature of *self-direction-thought* were acts concerned with self-development (e.g., buying and reading books, learning a skill, studying, reading poetry, attending a lecture) and creativity and aesthetics (writing poetry, playing a musical instrument, visiting a museum, or an art exhibition). The similarity of these behaviors to the value-expressive behaviors for self-direction-thought in previous research (Schwartz and Butenko, 2014) suggests that these too are value-expressive. Most of these behaviors also correlated negatively with values from the opposite side of the circle, suggesting that a value trade-off guides them. The core goal of *self-direction-action* values is to maintain freedom to determine one's own behavior. The observed pattern of correlations with behaviors is largely negative. The negative correlations with religious practices, gardening, donating, and changing one's environmental behavior comports with the motivation to resist outside pressures to perform conventionally expected acts. The value signature of self-direction-action values in these behaviors, while meaningful, is relatively weak. Other values related more strongly to these behaviors in several cases.

*Stimulation* values exhibited a clear, though not very strong, signature in a set of acts that involve excitement, novelty, and risk. Positive correlations with drinking to the point of intoxication and a hangover, betting and gambling, downloading and trading music, partying, and trying something completely new all comport with the goals of stimulation values. Negative correlations with all the religious practices suggest that these behaviors are usually perceived as unexciting. *Hedonism* values motivate pursuit of sensual pleasure. They exhibited a meaningful if weak positive signature in acts of drinking alcohol, trading music, partying, and gambling. They exhibited a somewhat stronger negative signature in their negative association with religious practices.

#### Self-Enhancement Values

People usually pursue the core goal of *achievement* values, success according to social standards, in work or academic contexts. None of the acts in the list of behaviors referred to these contexts. Not surprisingly, therefore, none of the acts reflected a clear achievement value signature. Perhaps a concern with being admired for success according to social standards is reflected in the positive correlations of achievement with online shopping behaviors (e.g., examined the clothing categories on eBay). None of the acts in the list described behaviors through which people might attain the core goal of *power-dominance values*, exercising control over others. However, the signature of this value did appear in its consistent though small negative correlations with six routine housekeeping activities (e.g., ironing clothes, making beds, etc.), activities often associated with positions of low power. Hints of the influence of power-dominance values are also found in its weak positive correlations with acts that may express a desire to influence others (attend a town meeting and donate to a political cause), to defy social conventions (become intoxicated, have a hangover), and to be involved with the stock market.

The core goal of *power-resources* values is control of material and social resources. Only the finance/investment acts in the list might relate directly to attaining resources, but they did not. Examination of the behavioral items that correlated with power-resources values in previous studies (e.g., Schwartz and Butenko, 2014) revealed that all referred to having or showing off one's wealth and none to taking actions that generate resources. This suggests that power-resources values are less concerned with acting to attain resources (e.g., investing) than with enjoying those resources. The signature of power-resources values did appear in negative correlations with religious and environmental activities and positive correlations with drinking, gambling, and partying. Aspiring to power-resources goes hand in hand with rejecting activities that provide no material benefits or may demand self-sacrifice and with embracing self-indulgent activities.

*Face* values motivate protecting one's public image and avoiding humiliation. None of the acts in the list described behaviors directly relevant to this goal and there was no evidence of a clear behavioral signature. All correlations with behavior were weak and negative. The only hint of the influence of face values was their negative correlations with going out alone to public entertainment (movies, concert, or theater), acts that might be embarrassing if done alone.

#### Conservation Values

*Security-personal* values concern maintaining certainty and protecting oneself from threat or danger. Their correlations were all negative and weak, indicating no clear behavioral signature, perhaps because none of the behaviors explicitly referred to either self-protective or highly dangerous acts. These values did, nonetheless, correlate negatively with acts that might be experienced as entailing some risk, whether of social failure (performing music in public and writing or reading poetry [publically]) or physical injury (running or jogging, boating or rafting). *Security-societal* values concern the safety, well-being, and protection of societal institutions. These values exhibited a weak but consistent signature through positive correlations with acts favorable to societal institutions and the general welfare, engaging in the practices of religion, donating to charity, and conserving natural resources (e.g., using both sides of a paper).

Unsurprisingly, *tradition* values had a strong signature in the religious practices. These values also correlated positively with some of the homemaking activities and with traditional behaviors such as working on a scrapbook and writing a thank-you note.

*Conformity-rules* values motivate compliance with rules and formal obligations. The behavioral signature of these values, though weak, was consistent with their core goal. They correlated negatively with socially disapproved drinking behaviors and gambling with cards or dice. Especially appropriate were two acts that correlated only with conformity-rules values, working on a retirement plan—positively and drinking during working hours—negatively.

*Conformity-interpersonal* values motivate avoiding upsetting or hurting people with whom one interacts. None of the acts in the list described behaviors directly relevant to this goal and there was no evidence of a clear behavioral signature. Consistent with behaving in ways that do not disturb others, conformity-interpersonal values had weak positive correlations with a few religious practices and some weak negative correlations with drinking behavior.

*Humility* values express an understanding that one is insignificant in the larger scheme of things. This leads to avoiding overvaluing the self, boasting, or publicly asserting one's worth. None of the acts in the list was directly relevant to expressing humility values. However, hints of their signature may be present in their negative correlation with behaviors that people who may consider themselves particularly socially skilled or attractive are more likely to perform: dating, partying, participating in an online discussion group, using an mp3 player or an iPod, and examining clothing choices on eBay. Humility values also correlated positively with some religious practices and negatively with some drinking behaviors, consistent with valuing self-restraint.

#### Self-Transcendence Values

*Universalism-nature* values exhibited a clear behavioral signature in two sets of behaviors, those concerned with protecting or enjoying nature (e.g., changing a habit to reduce impact on the environment, composting, gardening, going to an animal show, fishing, or hunting) and those concerned with art and aesthetics (e.g., reading and writing poetry, producing a work of art, reading a fashion related book). Such acts express both the central goal of preserving nature and the closely related goal of appreciating and promoting beauty (Schwartz, 1992). *Universalism-concern* values had no strong or unique associations with behavior. It correlated most positively with religious practices and homemaking, and negatively with drinking alcohol and gambling, reflecting a conventional, religious orientation. The lack of a clear behavioral signature may be due to the absence of acts relevant to its core goal of equality and justice for all people. *Universalism-tolerance* values also had no behavioral signature in the list of acts. All its correlations were <0.14. For this value too, the list lacked acts relevant to its core goal—accepting and understanding those who are different from oneself.

Neither *benevolence-caring* nor *benevolence-dependability* values had clear behavioral signatures. There were no substantial positive correlations. This is not surprising because none of the acts in the list of behaviors express the shared goal of the two benevolence values, promoting the welfare of one's family and friends. Five of the 10 most highly correlated acts were the same for both values, and all five correlations were negative and weak. Nonetheless, these correlations were consistent with what benevolence values might motivate one to do: avoiding activities that might entail some risk to the welfare of one's family (e.g., political involvement, going to a casino) or that might reflect undue self-preoccupation (e.g., reading a fashion-related book). Moreover, benevolence-dependability values correlated negatively with acting irresponsibly in ways that might undermine confidence in people's reliability (e.g., drinking, riding a motorcycle).

#### Raw vs. Centered Value Scores

The correlations obtained between the frequency of behavioral acts and both raw and centered value scores were largely similar. They differed somewhat more for self-direction-action, achievement, power-dominance, conformity-rules, humility, benevolence-caring, and benevolence-dependability. The raw value scores exhibited a larger number of positive and a smaller number of negative correlations than the centered scores did. In several cases, raw scores correlated positively with seemingly irrelevant behaviors, but the corresponding centered scores did not. In other cases, raw scores failed to correlate negatively with a behavior they were expected to oppose, but the corresponding centered scores did. For instance, consider self-direction-action values. Contrary to expectations and past findings (e.g., Schwartz and Butenko, 2014), the raw score correlated positively with shopping but failed to correlate negatively with religious practices whereas the centered scores correlated as expected. As another example, consider benevolence-caring. Its raw score correlated positively with the irrelevant “studying some subject” but its centered score did not. On the other hand, its raw score failed to correlate negatively with finance and investment activities. The results based on centered scores are usually more informative because they more often reveal behaviors that values may inhibit, not only behaviors that values may promote.

#### Summary of Findings

The correlational analyses conducted on 19 basic values and 209 ORAIS items revealed clear behavioral signatures for four values: self-direction-thought, stimulation, tradition, and universalism-nature. Six other values exhibited weak behavioral signatures, primarily in negative correlations that suggested that these values inhibited particular behavioral acts: self-direction-action, hedonism, power-dominance, power-resources, societal security, and conformity-rules. The remaining nine values showed no noticeable behavioral signatures in the everyday acts we studied.

Some of the correlations found in this study provide new insight into relationships between value preferences and behaviors. For instance, previous research linked self-direction-action values to behaviors that directly reject non-specific outside pressures (e.g., “Do something my way even if someone might disapprove”; Schwartz et al., 2012). The negative association of self-direction-action values with engaging in religious practices in the current study revealed that these values may also be expressed in self-assertion in the face of specific conventional expectations. Other examples of new insights into the domains of application of particular values include evidence that benevolence-dependability values relate negatively to irresponsible behaviors (e.g., becoming intoxicated), humility values relate negatively to acts performed more frequently by self-confident people (e.g., dating, partying, and participating in online discussion groups), and face values relate negatively to potentially embarrassing activities (e.g., attending public entertainment like movies alone).

#### Limitations

The sample in this study was not representative of the population, although it was quite heterogeneous in its demographic characteristics. Most prominently, the sample was skewed toward younger adults (*M* = 29.72, *SD* = 12.64). Hence the distributions of values and behaviors do not capture their actual occurrence in the adult population. One should keep this in mind when generalizing about value-behavior relations from this study.

The findings of this study were strongly affected by the limited range of measured behaviors. We chose to use the ORAIS items because they claimed to provide broad coverage of the domains of everyday behavior. However, some crucial domains were underrepresented or missing in the ORAIS pool of behaviors. There was a dearth of behaviors in the occupational and educational domains. Nor were there behaviors of cooperative or conflictual social interaction. This limited our ability to discover the behavioral signatures of several values. Achievement values are most frequently expressed in work and educational settings. Benevolence-caring, benevolence-dependability, universalism-concern, universalism-tolerance, conformity-interpersonal, face, humility, and security-personal behaviors are most frequently expressed in social interaction settings. Consequently, we were able to identify weak behavioral signatures of these values at best. Future research should utilize a larger and more diverse pool of behavioral acts in order to identify the value signatures of the 19 basic values in everyday behavior.

The use of self-report measures of values and behavior made this study vulnerable to self-presentation and consistency biases that might enhance value-behavior correlations. Within-person centering of the values partly reduced bias because correlations were then based on person's value hierarchies rather than the absolute importance they ascribed to values. Centering also led to the identification of more negative and meaningful value-behavior associations. Self-presentation may be less likely to generate negative correlations. Separation of the value and behavior measurement by 2 weeks weakened possible consistency biases.

Finally, our use of retrospective measures of behavior frequency may have introduced recall bias. Goldberg (2010) argued that the method we used reduces recall bias. He noted that people are quite accurate in differentiating between frequent activities (>10 times a year) and those they enacted rarely if ever, even when they cannot say exactly how often they engaged in an activity. He designed the response scale for the ORAIS behavior list we used in order to maximize accuracy. However, this scale may not be suited to capture the range of variation in extremely frequent or infrequent behaviors. Alternative response scales that assess frequency relative to opportunities to perform a behavior (e.g., Schwartz and Butenko, 2014) might be more appropriate.

## Study 2

Study 2 addressed some of the limitations of Study 1. Study 2 sampled behavioral acts from people's real-time reports of what they were doing in the last few minutes. We related these acts to participants' self-reports of value states (i.e., the values activated in the specific situation). Obtaining real-time reports reduced the risk of recall bias. Sampling actual on-going behavior permitted capturing the full range of activities in which participants engaged. Like Study 1, also Study 2 was exploratory. So we formulated no specific hypothesis regarding possible associations between value states and everyday activities.

### Participants

Participants were 374 Polish individuals, aged 17 to 53 (*M* = 23.72, *SD* = 4.67), 79% females, all Caucasian. Paid research assistants recruited participants to take part in a three-wave study including an online survey and experience sampling. Research assistants recruited participants in two ways: (a) from their close and distant relatives, friends, and acquaintances, and (b) from respondents to advertisements published in social media. Participants received a voucher of 70 PLN (~20 USD) after completing the three waves of data gathering. The current study utilized data only from the first wave^1^. The study was confidential—the research assistants had no access to any survey or experience sampling data and the researchers had no access to participants' personal data. Participation was voluntary.

The study received ethical approval from the Commission of Ethics and Bioethics at Cardinal Stefan Wyszyński University in Warsaw. All participants provided informed oral consent.

### Procedure

Participants downloaded an experience sampling mobile app (RealLife Exp) to their own mobile devices. For the next 7 days after activating the app, participants received a prompt seven times per day, at random times between 9.30 a.m. and 9.30 p.m. There was a minimum of 60 min between prompts. The prompts provided a link to a set of questions that participants were asked to answer. If they did not respond within 45 min, the link became unavailable. Each question appeared on a separate screen. Responding to the questions did not require an internet connection. Responses were sent to the server once the participant's device was connected to the internet.

### Measures

The experience sampling form contained 16 questions (including five closed-ended questions, not relevant to this study). A first, open-ended question asked, “What have you been doing during the past 15 min?” The answers to this question yielded a pool of everyday activities. The second question asked whether the activity was volitional or not. It presented the following two options: “This activity was imposed by another person or by the circumstances” or “This activity was my choice—I could either do or not do it.” The next nine questions measured different value states. Each of them started as follows: “When you were engaging in this activity, how important was it to you to…?” The endings of this question represented nine values differentiated in the refined version of the Schwartz value theory (Schwartz et al., 2012; Schwartz, 2017). The items also represented nine of the values from the original 10 basic values (except tradition, which we excluded based on the assumption that people pursue it infrequently in everyday behavior). Table 3 presents the nine values and the endings provided in the respective question. The value questions appeared one at a time, in random order. Participants responded on a scale from 1 (*not important at all*) to 4 (*very important*).

**Table 3:** Items measuring value importance in real-time behavior (Skimina et al., 2018a).

**Value**	**When you were engaging in this activity, how important was it to you to**
Self-direction-thought	- Understand something or form an opinion on your own
Stimulation	- Experience something new or exciting
Hedonism	- Enjoy yourself
Achievement	- Be better at something than others are
Power-resources	- Gain some advantage for yourself
Security-personal	- Avoid danger
Conformity-interpersonal	- Do what someone else expected
Universalism-concern	- Help someone you did not know
Benevolence-caring	- Help people you care about

### Pool of Behaviors

Participants reported 13,873 behavioral acts in total. They described 9,592 of these as volitional, 4,254 as non-volitional, and did not classify 27. In this study we analyzed only the behaviors described as volitional because we assumed that non-volitional acts do not reflect one's true values. We excluded 97 responses that reported that all nine values were very important for an act. The content of these responses had no common theme; the most frequent categories were working 17.5%, eating 11.3%, resting 9.3%, and traveling in a vehicle 7.2%. In addition, we excluded 79 responses either because they did not vary within person (all open-ended responses provided by the participant were identical) or because the open-ended response was meaningless or did not describe a behavior. After dropping these 176 responses, the final pool of behaviors included 9,416 voluntary acts provided by 369 participants, averaging 25.52 acts per person (ranging from 1 to 45).

### Analyses

In order to identify the behavioral signatures of values, we performed the analysis in three steps: First, we identified the pool of behavioral acts that were most related to value states. Second, we classified these behavioral acts into a smaller number of behavioral categories. Third, we identified the categories of behavioral acts that related most strongly to each specific value state.

#### Identifying the Pool of Behavioral Acts Most Related to Value States

The data set included up to 49 responses per person (7 per day × 7 days). Some respondents rated values as very important (4) for many behaviors whereas others used “4” only occasionally. This meant that the people who tended to use “4” more frequently would have greater weight in determining the values that were most important for behaviors. In order to reduce this source of bias, we person-centered all responses to the value items. For all participants, we calculated the mean importance rating he or she gave to the nine values across all of their behaviors. We then subtracted this mean from the importance rating they gave to each value state. This transformed the importance ratings that participants had made on the 1–4 scale to within-person centered scores. We then selected the behavioral acts with the highest scores for each value state across individuals. As a threshold for selecting the acts that related most strongly to each value state, we used a score of two standard deviations above (>2SD) the mean importance assigned to this value across all acts.

There were 565 acts that met the >2SD threshold for achievement, 329 for security-personal, 419 for conformity-interpersonal, 449 for benevolence-caring, 542 for universalism-concern, 232 for self-direction-thought, and 285 for stimulation. No acts met this criterion for power-resources and hedonism values. For these two value states, we selected the 5% of behavioral acts with the highest importance ratings attributed to these values. This yielded 547 acts for power-resources and 546 for hedonism. In this way, we obtained a grand pool of 3,914 behavioral acts across the nine values. Some acts were associated with more than one value state; hence, the total was less than the sum of the acts associated with the nine value states. These acts were provided by 331 individual participants (88.5% of the sample).

#### Classifying the Pool of Behavioral Acts Into Behavioral Categories

The grand pool of 3,914 acts was still too large to permit analyzing each act separately. We therefore combined the acts into categories based on their similarity. We first grouped together act descriptions that were nearly identical but differed in their tense or gender. This reduced the total pool to 646 behavioral acts. This pool of 646 behavioral acts is an empirically derived set of behaviors that individuals saw as most strongly related to value states in everyday life. We then asked two judges who were not familiar with the aims of the study (a psychology undergraduate student and a psychology PhD student) to further reduce this pool by grouping similar behaviors together to produce ~100 categories. We instructed them as follows:

You may group descriptions of two behaviors (labeled X and Y) into one category (Z) if using the label Z instead of X or Y causes no substantial change in the meaning of (a) the way the person performed the behavior (what the person did, the action taken, expressed by the verb used) and (b) the purpose of the behavior (why the person did it, the person's main aim, if one or more of the descriptions included a purpose).

The two judges worked together, following these instructions. They categorized 646 cards with the descriptions of behavioral acts 88 categories of everyday behavior. We used this set of 88 behavioral categories to identify the categories of everyday behavior that relate most strongly to each value state.

#### Identifying the Behavioral Categories Most Strongly Related to Each Value State

For each value state, we counted the number of behavioral acts strongly related to that value state that were classified in each category. This enabled us to identify the categories of behavior that participants linked most strongly to each value state, that is, the behavioral signature of each value state.

We also adopted a second perspective on behavioral signatures. In this perspective, we considered only the categories of behavior that participants rated as most strongly related to value states in general. For each behavioral category, we identified the value states that participants experienced most frequently as very important. Thus, for each category of behavior, we examined whether any specific value state exhibited a behavioral signature particularly strongly.

### Results and Discussion

Table 4 provides the data to identify the behavioral signatures of value states in response to the question: Which categories of behavior did participants most frequently link to each value state? The heading under each value state lists in parentheses the number of behavioral acts that were most strongly related to that value state (i.e., those acts with the highest scores for each value state across individuals). The cells in the column for each value state list the percent of the total acts strongly related to the value state from the behavioral category in the row. The percentages in each column sum to ≈100%. Thus, for example, of the 565 behavioral acts for which achievement values were considered highly important, 18.2% involved preparing for school, 11% involved working, and 10.3% involved physical activity.

**Table 4:** The distribution of behavioral categories for which the value states were very important in % (blank = zero; each column sums to 100%).

**#**	**Category of behavior**	**SDT (232)**	**ST (283)**	**HE (546)**	**AC (565)**	**POR (547)**	**SEP (329)**	**COI (416)**	**UNC (542)**	**BEC (449)**
**LEARNING—SCHOOL**										
1	Participating in school lessons								0.2	
2	Preparing for school individually	9.9	2.8	0.5	18.2	4.8	2.4	6.7	6.3	1.1
3	Exams		0.4					0.2		
**LEARNING—UNIVERSITY**										
4	Participating in lectures/seminars	10.3	5.3		7.6	3.5	2.4	5.0	3.9	0.4
5	Learning individually	0.4	0.4		2.1	0.4		0.7	0.2	0.7
6	Learning in group				0.9			0.5	0.6	
7	Exams		0.4		1.1	0.5		0.5		
**EXTRA CLASSES**										
8	Participating in extra classes	1.7	1.8		2.1	1.3	0.3	1.0	0.6	0.2
9	Preparing for extra classes individually	1.3	2.1	0.9	4.2	1.1		0.7	0.4	0.2
10	Exams						0.3	1.7	0.2	
**WORK**										
11	Working	8.2	4.2	0.7	11.0	3.1	8.8	21.9	26.8	5.3
12	Looking for a job				0.4	0.5	0.3		0.2	
**FAMILY LIFE—CHILDREN**										
13	Caring for a child (e.g., preparing meals)		0.7	1.3	0.2	0.2	1.2	1.4	0.9	4.0
14	Training cognitive skills with a child							0.5		0.2
15	Training physical skills with a child									0.4
16	Playing with a child			0.2	0.7	0.0	1.8	0.7	0.2	0.7
17	Participating in child-related events (e.g., school ceremonies)		0.4	0.2						0.2
**PETS**										
18	Walking pets						0.3			
19	Caring for pets					0.2				0.2
20	Playing with pets		0.4	1.1	0.2			0.7		1.6
21	Training pets			0.2						
**MAINTENANCE—PHYSIOLOGICAL NEEDS**										
22	Eating	3.9	3.5	5.5	2.7	12.2	7.3	4.8	6.3	6.9
23	Sleeping	0.4	1.4	3.7	0.7	9.7	4.9	0.5	1.5	0.2
24	Personal care	0.4		1.6	0.5	3.8	1.5	1.0	0.2	0.2
**MAINTENANCE—HOUSEKEEPING**										
25	Cleaning a house, washing clothes	0.4		0.5	1.9	1.8	2.4	4.3	1.7	7.6
26	Cooking	0.9	5.7	2.6	3.7	6.8	4.9	5.3	4.1	12.2
27	Repairing				0.2		0.9	0.5	0.4	1.1
**MAINTENANCE—ERRANDS**										
28	Formal (e.g., bank, post)	2.2	1.1	0.2	1.1	0.9	0.9	1.4	1.5	0.7
29	Informal (e.g., booking beauty salon appointment)					0.2				0.2
30	Medical (e.g., seeing a doctor)	0.4		0.2	0.2	0.5	1.5	1.0	0.4	0.7
**SHOPPING**										
31	Shopping online		0.4	0.4	0.2	0.2		0.5	0.2	0.2
32	Shopping	2.2	2.1	2.6	2.5	5.1	6.4	2.4	4.6	6.2
33	Buying presents online	0.9	1.1	0.5	0.2	0.4		0.2		1.3
34	Buying presents				0.2			0.5	0.2	0.4
**LEISURE—OTHER**										
35	Participating in sport or cultural events (e.g., concert, play, football game)	0.9	1.4	0.9		0.4	0.3	0.5	0.4	0.2
36	Having beauty treatment at a salon		2.1	0.7	0.5	2.0	0.3	0.5	0.2	0.4
37	Doing beauty treatment at home			0.4		0.2				
38	Getting ready to go out	0.4	0.4	0.4	2.8	1.6	0.3	1.2	0.9	0.9
39	Preparing for a date		0.4	0.2						
40	Preparing for a party			0.4	0.2	0.2				
41	Drinking alcohol	0.4	2.8	1.6	0.2	0.5			0.4	
42	Physical activity	4.3	9.9	7.7	10.3	9.5	11.6	4.1	4.4	3.1
**LEISURE—SELF-DEVELOPMENT AND RELAX**										
43	Reading	8.6	5.3	4.8	2.8	3.8	0.3	1.0	0.7	0.4
44	Reading/watching news	1.7			0.4				0.2	0.2
45	Reading about one's hobby	1.3	1.1	0.4	0.5	0.9			0.2	
46	Listening to music	1.7	4.2	3.3		1.6	0.6	0.7	0.0	0.4
**LEISURE—GAMES AND HOBBIES**										
47	Active games (e.g., pool, bowling)			0.2					0.2	
48	Puzzles, crosswords		0.4	0.4						
49	Board games	0.4	0.7	0.9	0.4		0.3	0.2		0.2
50	Computer games		1.8	7.1	4.8	1.1	0.9	0.7	1.7	0.7
51	Creative hobby (e.g., playing instruments)	2.2	3.5	0.5	1.9	0.5	0.6	1.9	1.1	2.7
**LEISURE—RESTING**										
52	Passive resting (e.g., lying on the sofa)		0.4	4.0	0.4	4.9	4.6	1.0	0.4	3.8
53	Watching TV (except news)	10.3	14.1	22.5	0.7	4.9	1.8	1.7	1.5	3.1
54	Using internet (except communication)	5.6	4.2	3.8	1.4	2.7	1.5	1.4	1.5	1.3
55	Thinking								0.4	0.2
**TRAVELING**										
56	Sightseeing		0.4	0.2	0.4		0.3		0.4	0.2
57	Hiking								0.2	
**SOCIAL INTERACTION FACE-TO-FACE**										
58	Talking with a family member	0.4	0.7	0.2		0.2	0.0	0.7		1.6
59	Talking with a partner		0.4	0.2		0.2	0.3	0.2		1.1
60	Talking with friends		0.4	1.3	0.2		0.3		0.2	0.9
61	Talking with acquaintances			0.4				1.2	1.7	0.7
62	Talking with strangers	5.2	2.5	1.6	1.1	0.4	0.9	2.4	2.6	3.8
**SOCIAL INTERACTION ON PHONE OR INTERNET**										
63	Talking with a family member									0.2
64	Talking with a partner							0.2		0.2
65	Talking with friends	0.9		0.2						0.7
66	Talking with acquaintances	2.2	0.4	0.7	0.5			2.6	1.8	1.3
**SOCIAL INTERACTION—SPENDING TIME WITH OTHERS**										
67	Hosting a family member	0.0	0.4	0.5	0.2			1.0	0.2	0.9
68	Hosting a partner									
69	Hosting friends									
70	Hosting acquaintances		0.4	0.7		0.2				0.2
71	Meeting a family member in a public place						0.3		0.4	0.2
72	Meeting friends in a public place		0.4	1.3		0.2		0.2		0.4
73	Meeting acquaintances in a public place	3.0	2.8	4.2	1.2	1.3	2.1	2.4	3.1	2.7
74	Visiting a family member		0.4	0.5	0.2	0.2	0.3	0.5	0.6	1.8
75	Dating	0.4	0.4	0.9	0.2	0.4	0.6	0.5	0.4	1.3
76	Participating in one's family events (e.g., wedding)			0.2						
77	Participating in others family events				0.2		0.3		0.4	0.7
78	Participating in meetings of social organizations (e.g., scouting)	0.9		0.2	0.2				0.4	
79	Working as a volunteer	0.4		0.4	0.4	0.2	0.3	0.7	1.3	0.2
80	Helping acquaintances	0.4					0.3	1.2	1.1	2.2
**OTHER**										
81	Waiting				0.2			0.2	0.4	0.7
82	Participating in contests. Surveys, etc.	0.4		0.4	0.4	0.4		0.5	0.4	0.0
83	Planning	0.4		0.2	0.4	0.4		0.2		0.2
84	Sexual activity	0.4	0.7	1.1	1.1	0.9	0.9	1.4	0.7	0.7
85	Traveling by car, bus, tram. train or plane	1.3	1.8	0.7	2.8	1.8	20.7	2.4	4.1	4.0
86	Religious practices		0.4	0.2	0.2	0.4	0.6	0.5	2.2	1.6
87	Other	0.4		0.2	0.2	0.2		0.2	0.4	0.2
88	Unclassifiable	0.9	1.1	0.4	0.4	0.5		1.0	2.2	

*The numbers in parentheses in column heads specify the number of behavioral categories for which that value state was mentioned as highly important*.

*SDT, self-direction-thought; ST, stimulation; HE, hedonism; AC, achievement; POR, power-resources; SEP, security-personal; COI, conformity-interpersonal; UNC, universalism-concern; BEC, benevolence-caring*.

#### Behavioral Acts Representative for Value States

##### Openness to change values

The real-time behaviors that participants most frequently linked to the *self-direction-thought* value state were self-development (participating in lectures/seminars and preparing for school) and resting (watching TV and reading). The self-direction-thought state was operationalized in this study as trying to “understand something or form an opinion on your own.” This is relevant for all these activities. Watching TV, categorized as passive resting, may serve not only for entertainment, but also for cultivating one's own opinions on various topics. For the *stimulation* value state, the most characteristic real-time behaviors were watching TV and physical activity. Other activities that participants linked to stimulation were creative hobbies, reading, and using the internet. They represent exciting activities conducted routinely on a daily basis. For the *hedonism* value state, the most characteristic real-time behaviors were watching TV, physical activity, playing computer games, and eating. These activities are all likely to be performed for pleasure. Drinking alcohol was not mentioned frequently as a real-time behavior. To the extent it was mentioned, however, it was related to stimulation and hedonism more than to other values, in line with the definitions of the values.

##### Self-enhancement values

The real-time behaviors that participants most frequently linked to the *achievement* value state were preparing for school individually, participating in lectures/seminars, working, and physical activity. These activities provide opportunities to pursue the goal of achievement values as operationalized in this study, “to be better at something than others are.” For the *power-resources* value state, the most characteristic real-time behaviors were eating, sleeping, physical activity, and cooking. In this study, we operationalized the goal of power-resources as “gain some advantage for yourself.” These behaviors could be understood as advantageous for oneself, although they do not express the conception of power-resources in the theory (“power through control of material and social resources”; Schwartz, 2017, p. 54). See the elaboration of this point in section Values perceived as the most important for various categories of behavioral acts.

##### Conservation values

The real-time behaviors that participants most frequently linked to the *security-personal* value state were traveling (by car, bus, tram, train, or plane), physical activity, working, eating, and shopping. Each of these may provide a sense of personal security and may be done for that purpose. Traveling in a vehicle might be accompanied by concerns with danger. Engaging in physical activity may be done in order to maintain fitness and health; it may sometimes also be experienced as dangerous. Working to earn a living can be critical to preserve security, and both shopping and eating often serve to provide the sustenance necessary for personal security. For the *conformity-interpersonal* value state, the most characteristic real-time behaviors were working, cooking, preparing for school, and attending lectures. We operationalized conformity-interpersonal as “to do what someone else expected.” These are activities in which one may be acting to please or meet other expectations—those of peers, teachers, and family members.

##### Self-transcendence values

The real-time behaviors that participants most frequently linked to the *benevolence-caring* value state were cooking, cleaning the house, eating, and shopping. Most of these activities directly serve the theoretical motivation of this value to promote the welfare of family and friends. Eating together with family or friends may be experienced as an expression of solidarity and mutually supportive closeness. For the *universalism-concern* value state, the most characteristic behaviors were working, preparing for school, and eating. Its operationalization was “to help someone you did not know.” Work may sometimes provide opportunities to help strangers, but that is an unlikely to be a frequent motivator of work. Nor does it make much sense that concern for others outside one's in-group is important for the other characteristic behaviors of universalism-concern. This was the value mentioned least frequently as very important in real-time behaviors. In the next section, we suggest another interpretation of findings for this value.

#### Values Perceived as the Most Important for Various Categories of Behavioral Acts

Table 5 identifies behavioral signatures of value states in response to the question: Which value states did participants experience as more and less important for the most common categories of behavior? Table 5 lists the 17 categories of behavior (of the 88 in Table 4) that included at least 50 single behavioral acts that participants linked strongly to at least one value state. The last column lists the total number of acts included in each category of behavior. Each row lists the proportion of those acts that participants experienced as strongly linked to the value states heading each column. Proportions sum to ≈1.00 in each row. This table displays the values that were seen as more or less important for each category of behavior. For example (row 1 of the table), the value state most frequently mentioned as highly important for acts classified as watching TV (except news) was hedonism (0.49 of the mentions), followed by stimulation (0.16). For acts classified as preparing for school (row 2), achievement (0.43) was the most frequently mentioned value state. Note that the same participant could list more than one value state as highly important for each of his or her acts.

**Table 5:** Proportion of acts in each category of behavior strongly linked to each value state (for the 17 categories from Table 4 that included more than 50 acts).

	**Category of behavior**	**SDT**	**ST**	**HE**	**AC**	**POR**	**SEP**	**COI**	**BEC**	**UNC**	**# acts**
1	Watching TV (except news)	0.09	0.16	**0.49**	0.02	0.11	0.02	0.03	0.06	0.03	253
2	Preparing for school individually	0.10	0.03	0.01	**0.43**	0.09	0.03	0.12	0.02	0.14	238
3	Physical activity	0.04	0.10	0.15	**0.20**	0.18	0.13	0.07	0.06	0.08	283
4	Participating in lectures/seminars	0.17	0.11	0.00	**0.30**	0.13	0.06	0.15	0.02	0.15	143
5	Computer games	0.00	0.05	**0.40**	0.28	0.06	0.03	0.03	0.03	0.09	96
6	Cleaning house, washing clothes	0.01	0.00	0.03	0.12	0.11	0.09	0.19	**0.36**	0.10	94
7	Talking with strangers	0.15	0.09	0.11	0.08	0.03	0.04	0.13	**0.21**	0.18	80
8	Creative hobby (e.g., playing instruments)	0.08	0.17	0.05	0.18	0.05	0.03	0.13	**0.20**	0.10	60
9	Traveling by car, bus, tram, train, or plane	0.02	0.03	0.03	0.10	0.06	**0.44**	0.06	0.12	0.14	156
10	Cooking	0.01	0.08	0.07	0.10	0.18	0.08	0.11	**0.27**	0.11	205
11	Shopping	0.03	0.03	0.09	0.09	**0.19**	0.14	0.07	**0.19**	0.17	151
12	Sleeping	0.01	0.04	0.18	0.04	**0.49**	0.15	0.02	0.01	0.07	109
13	Meeting acquaintances in a public place	0.07	0.08	**0.23**	0.07	0.07	0.07	0.10	0.12	0.17	98
14	Using internet (except communication)	0.14	0.13	**0.22**	0.09	0.16	0.05	0.06	0.06	0.09	94
15	Eating	0.04	0.04	0.13	0.06	**0.28**	0.10	0.08	0.13	0.14	240
16	Passive resting	0.00	0.01	0.24	0.02	**0.30**	0.17	0.04	0.19	0.02	90
17	Working	0.05	0.03	0.01	0.15	0.04	0.07	0.21	0.06	**0.36**	403

*SDT, self-direction-thought; ST, stimulation; HE, hedonism; AC, achievement; POR, power-resources; SEP, security-personal; COI, conformity-interpersonal; BEC, benevolence-caring; UNC, universalism-concern. The largest proportion in each row is shown in bold*.

For physical activity (row 3), participants perceived several value states as highly important: achievement (0.20), power-resources (0.18), hedonism (0.15), and/or personal security (0.13). This set of behavioral signatures suggests three main types of motivation for physical activity. Participants engaged in physical activity in order to compete and demonstrate their abilities (achievement and power resources), to maintain or enhance their health (security-personal) and/or to derive pleasure (hedonism). By looking across each row, it is possible to identify the value states that participants perceived as motivating each category of behavior. Following the order of behaviors in the table, we next note the behavioral signatures of value states that make intuitive sense, given the motivations that the value states represent. We then examine apparent behavioral signatures that are surprising.

For participating in lectures/seminars, participants perceived achievement, self-direction thought, and/or universalism-concern as highly important. For playing computer games, hedonism and/or achievement were highly important. For cleaning house and washing clothes, benevolence-caring and/or conformity-interpersonal were highly important. For talking with strangers, benevolence-caring, universalism-concern, and/or self-direction thought were highly important. For creative hobbies (e.g., playing an instrument)—achievement and/or stimulation and/or benevolence-caring^2^. For traveling (in a car, bus, tram, train, or plane)—security-personal^3^.

If we ignore power-resources (discussed in the next paragraph), the behavioral signatures of value states in several other categories of behavior also make intuitive sense. For cooking and for shopping, benevolence-caring was mentioned frequently. For sleeping, hedonism and/or security-personal were mentioned frequently. For meeting acquaintances in a public place, hedonism was mentioned frequently. For using the internet (except for communication), hedonism and/or self-direction-thought and/or stimulation mere mentioned most frequently.

The apparent behavioral signatures of the power-resources value state, revealed when looking down its column, are often puzzling. The motivational goal that defines this value is “power through control of material and social resources” (Schwartz, 2017, p. 54). Examples of its correlates in value-behavior studies are “deciding to do a task or choose a job mainly based on the pay,” “showing off one's valuable possessions,” and “aspiring to be rich.” Here, the power-resources value state was the value most frequently mentioned as highly important for a set of behaviors that do not express the motivational goal of power resources: eating, shopping, sleeping, and passive resting. It was also highly important for cooking, physical activity, and using the internet. This set of behaviors might fit with the probe we used to operationalize power-resources in this study, “gain some advantage for yourself.” Participants may have interpreted this probe as referring to any behavior that was personally beneficial to them—physical benefit from eating, sleeping and resting, and benefit from the outcomes obtained by shopping, cooking, physical activity, and using the internet. The behavioral signatures of the probe, interpreted in this way, make good sense. But these are not behavioral signatures of the intended power-resources value state.

The apparent behavioral signatures of the universalism-concern value state were also somewhat puzzling. Participants mentioned this value as highly important for working (0.36), followed by the conformity-interpersonal and achievement value states. The latter states point to common reasons for working—meeting expectations and performing successfully. Less clear is why “help[ing] someone you did not know” should be the value state most frequently linked to working. Other behaviors for which universalism-concern received at least 15% of the mentions as highly important were talking with strangers, shopping, meeting acquaintances in a public place, and participating in lectures/seminars. Interestingly, the proportion of mentions in the column for universalism-concern in Table 5 correlated 0.68 with that for conformity-interpersonal. Its correlations with the proportions of mentions of the other value states were all <0.19. This suggests that participants understood this value state, as operationalized here, at least partly as “doing what one is expected to do (e.g., working).” Together, this meaning and the intended meaning fit the findings reasonably well.

#### Value-Expressive and Value-Ambivalent Behaviors

The evidence from the behavioral signatures of the values is consistent with the assertion that multiple values may propel most everyday behaviors (Bardi and Schwartz, 2003). For 14 of the 17 categories of behavior in Table 5, two or more different value states received mention as highly important for the behavior. This could result either from different people mentioning different value states as relevant for the behavior or from the same person mentioning more than one value state. Only three behaviors were clearly value-expressive in the sense that a single value state was mentioned for a large proportion (>0.4) of its acts and no other value state was mentioned frequently (e.g., watching TV [except news] expressed hedonism). The small number of clearly value-expressive behaviors is not surprising. In earlier value-behavior studies, researchers chose behaviors expected to be value-expressive. Here, in contrast, the behaviors studied were not selected for their value relevance but occurred in real-life settings.

Lönnqvist et al. (2013) suggested that some behaviors, which they labeled value-ambivalent, might express opposing values. These behaviors might be motivated by values on one side of the motivational circle of values for some people and by values on the other side of the circle for others. Alternatively, opposing values may motivate a behavior of a single person but on different occasions, not in the same, single act.

We were able to identify value-ambivalent behaviors in Study 2 because it examined participants' freely generated behaviors rather than behaviors chosen to be value expressive. The data revealed several such behaviors. Participants linked attending lecture/seminars (row 4 in Table 5) to values from around the whole circle. They linked sleeping (row 12) to hedonism on one side of the circle and to security-personal on the other side. They linked working (row 17) to achievement and to the opposing conformity-interpersonal^4^ value. Finally, they linked creative hobbies (row 8) to benevolence-caring and to the opposing achievement/stimulation values. The people who linked a behavior to values on opposing sides of the circle may have been engaging in different aspects of the behavior. For example, those who linked creative hobbies to benevolence-caring may have been singing in a chorus whereas those who linked hobbies to achievement/stimulation may have been engaged in woodwork or sports. Many of the other behaviors that participants linked to multiple value states were not value-ambivalent. Rather, the multiple value states linked to these behaviors were adjacent in the motivational circle and therefore compatible (e.g., rows 1, 3, 5, 6, 7).

#### Summary of Findings

Study 2 showed that many common, everyday activities have value signatures. The study identified specific real-time behaviors that were particularly expressive for each value state. For achievement, these behaviors included preparing for school, participating in lectures, working, and physical activity. For security-personal it was traveling by car, bus, tram, train, or plane. For conformity-interpersonal—working, for benevolence-caring—mainly housekeeping duties (cooking, cleaning), for universalism-concern—working, for self-direction-thought—participating in lectures and reading, for stimulation—creative hobbies, reading, and using the internet. For hedonism—watching TV and playing computer games, but also meeting with friends.

There was also evidence of a few value-ambivalent behaviors, sleeping for example, which was sometimes motivated by hedonism but at other times or for other people by the opposing security-personal. However, the many everyday activities that were linked to multiple value states were not value-ambivalent. Rather, they were related to two or more values that are adjacent in the motivational circle.

#### Limitations

Study 2, like Study 1 was based on a heterogeneous sample that was not representative of the population. Moreover, it was skewed toward younger adults. This necessarily affected the content and frequency of the behavioral acts that were mentioned and implies that care should be taken in generalizing our findings. The study also relied on self-reports, though sampling real-time behaviors minimized recall bias.

The study required installing a mobile app into participants' smartphones or tablets. This limited the sample to mobile device users. Moreover, technical problems in using the app caused some loss of data and increased variation in the number of responses provided by different participants.

Finally, the results of Study 2 suggest that two value states were poorly operationalized. Participants probably understood them in ways that failed to capture the conceptual meanings of the values they were intended to represent. Participants apparently understood power-resources as concerned with obtaining any benefit (e.g., even eating), rather than gaining power through material resources. They probably understood universalism-concern at least partly as doing what is expected rather than acting to promote the welfare of those beyond one's in-group. Future research on value states must develop operationalizations that capture these two value concepts more accurately.

## General Discussion

Our goal in the current studies was to identify everyday activities that have value signatures but that were not selected a priori as presumably value-expressive. We aimed to identify possible associations between basic human values and a wide variety of daily behaviors. We analyzed two sorts of data: (1) retrospective reports of the frequency of activities performed during the recent year and (2) real-time self-reports of activities performed during the past 15 min. We correlated activities recalled retrospectively with basic value traits measured as dispositions likely to guide behavior across time and situations. We associated activities reported in real time with reports of value states provided at the same time. Value traits refer to the importance individuals attribute to general motivations whereas value states refer to situational goals that guide specific real-time behaviors (Skimina et al., 2018a).

Study 1 examined relations of 19 basic human values with the frequency of 209 behavioral acts. We calculated partial correlations to control for gender and age. Although the correlations were mostly weak, they were consistent with theory. It is important to note that an approximate 2-week period elapsed between the measurement of value preferences and of the frequency of behaviors. The average correlations across the 10 most strongly associated behaviors for each value ranged from 0.11 (benevolence-caring) to 0.27 (self-direction-thought and tradition). These results are comparable to those in studies intended to identify behavioral signatures of the Big Five personality. Using the same pool of behavioral acts we used, Elleman et al. (2017) found that the average correlations across the 10 most strongly associated behaviors ranged from 0.18 for emotional stability to 0.36 for extraversion. Using a pool of 400 behavioral acts, Chapman and Goldberg (2017) reported that most correlations with the Big Five traits ranged from 0.20 to 0.30.

Although many correlations were weak, implying that the signatures of the values in those behaviors were weak, many of these correlations were in line with the theoretical expectations of the value circle. For instance, self-direction-action correlated negatively with a set of behaviors that imply a motivation to resist others' expectations to perform conventionally desirable acts (e.g., religious practices, donation, and changing one's environmental behaviors).

The findings from Study 1 also provided evidence for the Schwartz et al. (2017) suggestion that behaviors are products of value trade-offs. Many behaviors correlated positively with adjacent values in the motivational circle and negatively with opposing values. For example, drinking alcohol correlated positively with stimulation, hedonism, and self-enhancement values but negatively with conservation and self-transcendence values. Religious practices correlated positively with conservation values but negatively with openness to change and self-enhancement values.

Study 2 examined the associations of value states with a freely generated pool of everyday behaviors measured in real-time. The study obtained immediate, on-line evaluations of the importance of value states as guiding real-time behaviors. We focused the analysis on those behaviors for which participants reported the highest levels of importance of particular value states. Participants were free to choose any activity that they had engaged in during the past 15 min. The reports included common, daily activities. We examined to what extent value states were important for performing these activities. We found clear real-time behavioral signatures for some value states. For instance, the importance of security-personal was very high while traveling in a vehicle. Unsurprisingly, achievement was associated with learning and hedonism was associated with leisure activities.

Comparing Studies 1 and 2 revealed that some value-behavior associations emerged for both value traits and value states whereas others were limited either to value traits or to value states. For instance, participating in lectures related to self-direction-thought as both a trait (Study 1) and a state (Study 2). Similarly, religious practices related to universalism-concern as both a trait and a state. However, religious practices related to benevolence-caring only as a state (Study 2) but not as a trait (Study 1). This may reflect the different perspectives of the two studies. Study 1 sought to identify relations between the general importance people attribute to values and the frequency of many different recalled behaviors. The general value trait of benevolence-caring may rarely be relevant to the recall of religious practices, yielding a weak association. Study 2 identified the values activated at the time people engaged in a specific behavior. While actually engaged in a religious practice (e.g., praying with or for one's family members at church), the value state of benevolence-caring was experienced as relevant.

These findings highlight a major difference between the two approaches we adopted to study value-behavior relations. Correlations between value traits and the frequency of behavioral acts are most likely for value-expressive behaviors that many different people can perform. Saying grace at a meal, common in the venue of this study, is one example. Everyone has opportunities to perform this behavior, but only religious people are motivated to do so. For this reason, variation in the importance of tradition values relates strongly to the frequency of blessing at a meal. In contrast, value states experienced in real-time behavior, reflect not only the values that motivate the behavior but also the states that the behavior itself activates. For instance, tradition may motivate and be experienced as a value-state relevant to praying at church. The act of prayer itself may focus attention on loved ones, however, and thereby activate the value state of benevolence-caring. Thus, this behavior links prayer and benevolence-caring, but benevolence-caring rarely motivates prayer across people and situations, so the value trait relates weakly to prayer.

This difference between value traits and states helps to explain why some behaviors were related to many different value states in Study 2 though not in Study 1. For instance, the numbers of participants who mentioned achievement, hedonism, and security-personal value states as very important during physical activity was greater than the number who mentioned the stimulation value state. Yet, in Study 1, the stimulation trait correlated more highly with exercising, running, and jogging, suggesting that it was the more important motivator. While engaging in these sports, however, the activity itself may have activated the achievement and security-personal value states, because of its competitive and risk of injury aspects. Hence, we can interpret the results of Study 2 in two ways: (a) as indicating value states that motivate people to engage in everyday activities and/or (b) as revealing everyday activities that trigger/activate value states.

Applying Trait Activation Theory (Tett and Guterman, 2000) to values, we posit that individual differences in the importance of value traits lead to individual differences in the level of the corresponding value states people experience while engaging in relevant behaviors. For instance, compare the experience of the value state of personal security while driving a car for individuals who attribute high vs. low importance to the value trait of personal security. Those for whom the trait is highly important are more likely to experience the value state of personal security and/or to experience that state more strongly than those who attribute little importance to this trait. The former will be more motivated to drive more carefully. We can summarize this hypothetical model of relations between values and behavior as follows: (1) Situational cues activate relevant value states; (2) the level of the activated value state depends on the importance people attribute to the corresponding value trait; (3) the activated value state motivates relevant behavior. Future research can assess this model.

Boyd et al. (2015) extracted peoples' personal values and their recent behavior from open-ended essays in one study and from their Facebook status updates in another study. They also used a self-report questionnaire (the Schwartz Value Survey; SVS) to measure personal values. They compared value-behavior correlations obtained with the open-ended and questionnaire measures of values. As in our Study 1, Boyd et al. (2015) measured values as traits and they measured behavior retrospectively. As in our Study 2, they measured behavior with free response language data and grouped reported behaviors into categories. They found a larger number of significant associations of everyday behavior with values extracted from free response language data than with values measured with self-reports. All positive and negative correlations with self-reported values were consistent with the value definitions. However, many of the correlations with free response based values made little intuitive sense. We agree with Boyd et al. (2015) that using free response data to extract value traits from the vast corpus of available texts produced by individuals holds much promise. It may be necessary, however, to apply theory about the nature and structure of value systems to make sense of what is extracted.

Finally, Study 2 provided evidence that some behaviors are value-ambivalent (cf. Lönnqvist et al., 2013). That is, the same behavioral act was associated with opposing values for different people and/or for the same person on different occasions. For instance, both the achievement and the opposing benevolence-caring states were mentioned as important for creative hobbies. Most behaviors that were linked to multiple value states were not value-ambivalent, however. The multiple values that were activated were adjacent in the motivational circle of values, in other words, compatible with one another.

In sum, the current research demonstrated associations between values and a wider set of everyday behaviors than previously studied. The results of the two studies led to two major conclusions: (a) even common, everyday behavioral acts may be motivated by personal values and (b) value traits and value states may relate differently to those acts. The first conclusion provides a rational for further investigation of associations between everyday activities and values. In future research on value traits, a wider pool of behaviors should be measured. The pool of activities used in this study was too narrow to capture the behavioral expression of some of the values in the refined value theory (Schwartz et al., 2012; Schwartz, 2017). In future research on value states, improved operations are needed for two of the values and the 10 value states not included in the current research should be studied.

The second conclusion emphasizes the importance of distinguishing value traits from value states. In Study 1, trans-situational value traits, measured by questionnaire, related positively to behaviors likely to promote their goals and negatively to behaviors likely to hinder goal attainment. This method succeeded in identifying value trade-offs that may underlie behaviors. In Study 2, participants mentioned situation-specific value states that were important when engaging in behavioral acts. This method tended to focus respondents on the values they experienced as propelling their acts. As a result, it did not identify possible inhibiting value states that were overcome by the propelling value states when engaging in these acts. Thus, this study did not reveal the value trade-offs that often underlie behavior according to the value theory. Future research might seek to develop ways to identify possible trade-offs between opposing value states.

## Author Contributions

ES: study design, data analysis, data interpretation, manuscript preparation, literature search; JC: study design, data interpretation, manuscript preparation, funds collection; SS: data interpretation, manuscript preparation; ED and RA: manuscript preparation, funds collection.

### Conflict of Interest Statement

The authors declare that the research was conducted in the absence of any commercial or financial relationships that could be construed as a potential conflict of interest.

## Footnotes

^1^Skimina et al. (2018a) used the data from this study to test the circular structure and the importance hierarchy of value states. They did not use the data from the open-ended reports of behavior or examine relations of value states to behavioral acts, the focus of this study.

^2^Hobbies are frequently engaged in with friends.

^3^Apparently, traveling elicited concern about danger for a substantial subgroup of participants.

^4^As discussed earlier, universalism-concern, which also opposes achievement, may function like conformity here.
