# Supplementary material for: Behavioral Signatures of Values in Everyday Behavior in Retrospective and Real-Time Self-Reports
Source: Front Psychol. 2019 Feb 19;10:281. doi: 10.3389/fpsyg.2019.00281 (PMC6401649; doi:10.3389/fpsyg.2019.00281)
Supplement: Supplementary file 1 [file Data_Sheet_1.docx]

Table A

*Correlations between centered value traits and 89 behavioral acts*

| No | Item | Correlation | SDT | SDA | ST | HE | AC | POD | POR | FAC | SEP | SES | TR | COR | COI | HU | UNN | UNC | UNT | BEC | BED |
| --- | --- | --- | --- | --- | --- | --- | --- | --- | --- | --- | --- | --- | --- | --- | --- | --- | --- | --- | --- | --- | --- |
| *Children* | | | | | | | | | | | | | | | | | | | | | |
| 175 | Read the comics to a child. | Partial | .06 | -.05 | -.08 | -.09 | **-.16** | -.05 | -.07 | -.04 | .02 | .01 | .11 | .08 | .01 | .04 | .01 | .09 | .09 | .02 | .04 |
|  |  | Zero-order | .02 | -.09 | -.15 | -.15 | **-.22** | -.12 | -.13 | .00 | .11 | .03 | .12 | .14 | .05 | .05 | .08 | .15 | .14 | .07 | .07 |
| *Homemaking* | | | | | | | | | | | | | | | | | | | | | |
| 5 | Washed dishes. | Partial | .06 | -.02 | -.10 | -.14 | -.07 | **-.14** | -.13 | -.04 | -.02 | .08 | .11 | .05 | .04 | .04 | .01 | **.14** | **.13** | .04 | .09 |
|  |  | Zero-order | .03 | -.05 | -.15 | -.19 | -.12 | **-.19** | -.18 | -.01 | .05 | .10 | .13 | .10 | .07 | .05 | .07 | **.18** | **.17** | .09 | .12 |
| 9 | Cared for a potted plant. | Partial | .09 | -.05 | -.06 | -.06 | -.09 | **-.15** | -.12 | -.06 | -.03 | .03 | .01 | .05 | .07 | .01 | **.16** | .08 | .08 | .10 | .05 |
|  |  | Zero-order | .01 | -.12 | -.19 | -.19 | -.22 | **-.25** | -.22 | .00 | .14 | .07 | .05 | .16 | .12 | .04 | **.26** | .17 | .16 | .18 | .10 |
| 38 | Made a bed. | Partial | .02 | -.04 | -.04 | -.07 | -.01 | **-.17** | -.12 | .03 | -.03 | .03 | .07 | .04 | .06 | .05 | .01 | .10 | **.13** | .07 | .02 |
|  |  | Zero-order | .02 | -.04 | -.04 | -.06 | .00 | **-.18** | -.13 | .04 | -.02 | .01 | .06 | .04 | .06 | .04 | .01 | .10 | **.15** | .09 | .04 |
| 70 | Cleaned the house. | Partial | .09 | -.04 | .00 | -.08 | -.08 | **-.16** | -.13 | -.09 | -.02 | .02 | .09 | .01 | .02 | .06 | .05 | .09 | **.16** | .08 | .06 |
|  |  | Zero-order | .06 | -.08 | -.05 | -.12 | -.12 | **-.23** | -.19 | -.04 | .06 | .01 | .09 | .06 | .05 | .05 | .09 | .14 | **.22** | .15 | .12 |
| 103 | Ironed linens or clothes. | Partial | .05 | -.10 | -.08 | -.15 | -.04 | **-.17** | -.10 | -.10 | -.03 | .11 | **.20** | .09 | .05 | .05 | .01 | .12 | .09 | .05 | .08 |
|  |  | Zero-order | .02 | -.13 | -.14 | -.18 | -.10 | **-.26** | -.17 | -.05 | .07 | .09 | **.19** | .13 | .08 | .04 | .07 | .17 | .17 | .13 | .14 |
| 135 | Cooked a meal. | Partial | .07 | .01 | -.09 | -.09 | -.06 | **-.13** | -.14 | -.05 | -.02 | .07 | .11 | .09 | .00 | .05 | .05 | .08 | .09 | .04 | .06 |
|  |  | Zero-order | .04 | -.03 | -.12 | -.12 | -.10 | **-.20** | -.19 | -.01 | .05 | .05 | .11 | .12 | .02 | .04 | .09 | .12 | .16 | .11 | .12 |
| 167 | Baked a cake, pie, cookies, or bread. | Partial | .01 | -.09 | -.04 | -.08 | -.03 | -.10 | -.05 | **-.12** | -.04 | .04 | .08 | .07 | .05 | .04 | .07 | .04 | .05 | .06 | .05 |
|  |  | Zero-order | -.03 | -.13 | -.10 | -.12 | -.09 | -.21 | -.14 | **-.04** | .08 | .00 | .08 | .12 | .08 | .00 | .12 | .12 | .17 | .17 | .14 |
| *Gardening* | | | | | | | | | | | | | | | | | | | | | |
| 42 | Gardened. | Partial | -.03 | -.10 | .01 | -.06 | -.04 | -.04 | -.06 | -.08 | -.03 | .02 | .10 | .06 | -.01 | .03 | **.17** | .01 | .00 | .04 | -.02 |
|  |  | Zero-order | -.08 | -.13 | -.09 | -.16 | -.15 | -.07 | -.10 | -.06 | .07 | .09 | .13 | .14 | .03 | .08 | **.23** | .06 | .00 | .04 | -.03 |
| 74 | Did yard work. | Partial | -.06 | **-.13** | .00 | -.05 | -.08 | -.02 | -.05 | -.07 | .01 | -.02 | .09 | .07 | .03 | .05 | .12 | .03 | .00 | .06 | .00 |
|  |  | Zero-order | -.10 | **-.16** | -.08 | -.14 | -.16 | -.04 | -.09 | -.06 | .09 | .05 | .11 | .13 | .06 | .09 | .18 | .07 | -.01 | .05 | -.02 |
| 107 | Planted or transplanted a plant. | Partial | -.02 | -.10 | -.06 | -.13 | -.07 | -.06 | -.06 | -.04 | .04 | .01 | .07 | .05 | .08 | .05 | **.16** | .03 | .01 | .03 | -.01 |
|  |  | Zero-order | -.11 | -.18 | -.23 | -.30 | -.25 | -.14 | -.16 | .01 | .22 | .12 | .12 | .20 | .16 | .12 | **.28** | .13 | .05 | .07 | .00 |
| 171 | Bought plants for a garden or yard. | Partial | .02 | **-.12** | .03 | -.04 | -.04 | .03 | .00 | -.11 | .03 | -.08 | .07 | .04 | .04 | -.01 | .09 | -.03 | .00 | .01 | -.02 |
|  |  | Zero-order | -.07 | **-.19** | -.16 | -.22 | -.23 | -.08 | -.12 | -.04 | .22 | .04 | .12 | .19 | .12 | .06 | .22 | .09 | .06 | .07 | .00 |
| *Shopping* | | | | | | | | | | | | | | | | | | | | | |
| 122 | Ordered food to be delivered. | Partial | .03 | .01 | .08 | .12 | .09 | -.01 | .07 | -.03 | -.07 | -.07 | -.01 | -.03 | -.03 | **-.14** | -.02 | -.09 | .06 | .01 | .04 |
|  |  | Zero-order | .09 | .07 | .20 | .24 | .22 | .05 | .15 | -.06 | -.19 | -.15 | -.05 | -.14 | -.09 | **-.18** | -.13 | -.16 | .03 | -.02 | .04 |
| 130 | Purchased a musical album. | Partial | .16 | .06 | .10 | -.01 | -.04 | .04 | -.06 | -.07 | **-.14** | -.08 | .01 | .02 | -.03 | -.12 | .00 | .00 | **.13** | .01 | -.01 |
|  |  | Zero-order | .19 | .09 | .15 | .06 | .03 | .07 | -.02 | -.09 | **-.18** | -.11 | -.01 | -.03 | -.05 | -.14 | -.05 | -.04 | **.11** | -.01 | -.02 |
| 162 | Shopped in a music store. | Partial | .18 | .02 | .12 | .02 | -.04 | -.01 | -.07 | **-.13** | -.12 | -.06 | -.02 | -.03 | -.03 | -.06 | .05 | .02 | **.12** | .05 | .02 |
|  |  | Zero-order | .20 | .04 | .15 | .06 | .01 | .01 | -.04 | **-.14** | -.15 | -.08 | -.03 | -.06 | -.04 | -.07 | .01 | .00 | **.10** | .03 | .02 |
| 179 | Shopped on the web. | Partial | .16 | .08 | .03 | -.02 | .11 | .00 | .06 | -.03 | -.08 | .01 | .00 | -.02 | **-.15** | -.13 | -.02 | -.03 | .04 | .02 | .01 |
|  |  | Zero-order | .21 | .13 | .15 | .10 | .22 | .07 | .13 | -.07 | -.20 | -.06 | -.03 | -.12 | **-.19** | -.16 | -.12 | -.10 | .00 | -.03 | -.01 |
| 189 | Examined the clothing categories on eBay. | Partial | .03 | .02 | .12 | .08 | **.16** | -.02 | .12 | .09 | -.04 | .01 | -.01 | -.10 | -.11 | **-.14** | -.03 | -.09 | -.01 | -.05 | -.08 |
|  |  | Zero-order | .08 | .06 | .21 | .20 | **.27** | -.02 | .14 | .08 | -.13 | -.10 | -.05 | -.18 | -.15 | **-.20** | -.11 | -.12 | .03 | -.01 | -.01 |
| 206 | Used eBay to buy or sell something. | Partial | .13 | .08 | .06 | .03 | **.15** | .00 | .08 | -.01 | -.07 | -.03 | -.03 | -.05 | **-.15** | -.13 | -.01 | -.06 | .03 | -.02 | .02 |
|  |  | Zero-order | .17 | .13 | .16 | .14 | **.25** | .06 | .15 | -.04 | -.18 | -.09 | -.06 | -.14 | **-.20** | -.17 | -.10 | -.12 | .00 | -.05 | .01 |
| *Being alone* | | | | | | | | | | | | | | | | | | | | | |
| 49 | Went to the movies alone. | Partial | .11 | .05 | .08 | .00 | -.06 | .02 | -.05 | **-.15** | .00 | -.05 | -.06 | -.02 | .01 | .02 | .01 | -.01 | .09 | -.02 | .04 |
|  |  | Zero-order | .09 | .03 | .03 | -.04 | -.10 | .00 | -.07 | **-.14** | .04 | -.02 | -.05 | .02 | .03 | .04 | .04 | .01 | .10 | -.01 | .03 |
| 88 | Went to a concert or theater alone. | Partial | .20 | -.02 | .12 | -.04 | -.14 | .03 | -.07 | **-.12** | -.10 | -.05 | .00 | -.01 | .03 | .00 | .03 | .01 | .09 | -.02 | -.02 |
|  |  | Zero-order | .17 | -.04 | .06 | -.09 | -.18 | .01 | -.09 | **-.11** | -.04 | -.01 | .02 | .04 | .05 | .03 | .07 | .04 | .09 | -.01 | -.03 |
| *Drinking alcohol* | | | | | | | | | | | | | | | | | | | | | |
| 62 | Drank whiskey, vodka, gin, or other hard liquor. | Partial | -.03 | .02 | .12 | **.18** | .15 | .09 | **.19** | .05 | -.02 | -.10 | -.05 | **-.15** | **-.13** | -.11 | -.08 | **-.12** | -.03 | -.02 | -.06 |
|  |  | Zero-order | -.01 | .04 | .14 | **.18** | .16 | .15 | **.23** | .02 | -.07 | -.08 | -.05 | **-.17** | **-.14** | -.09 | -.10 | **-.15** | -.09 | -.08 | -.10 |
| 92 | Drank in a bar or night club. | Partial | .04 | .07 | **.17** | **.20** | .08 | .12 | .16 | -.02 | -.05 | -.07 | -.09 | **-.15** | **-.16** | **-.14** | -.05 | -.11 | .00 | -.04 | -.05 |
|  |  | Zero-order | .11 | .13 | **.29** | **.32** | .23 | .19 | .24 | -.06 | -.20 | -.14 | -.12 | **-.26** | **-.22** | **-.18** | -.17 | -.19 | -.05 | -.09 | -.07 |
| 125 | Became intoxicated. | Partial | .04 | .08 | **.21** | **.26** | .13 | **.14** | **.19** | .00 | -.08 | -.11 | -.11 | **-.19** | **-.17** | **-.15** | -.04 | **-.16** | -.02 | -.05 | **-.14** |
|  |  | Zero-order | .10 | .15 | **.31** | **.36** | .26 | **.23** | **.28** | -.05 | -.23 | -.16 | -.14 | **-.28** | **-.22** | **-.17** | -.15 | **-.24** | -.09 | -.12 | **-.16** |
| 157 | Had a hangover. | Partial | -.04 | .05 | **.18** | **.22** | .12 | **.13** | .17 | .03 | -.04 | -.11 | -.08 | -.12 | **-.14** | -.12 | .00 | -.12 | -.05 | **-.11** | **-.16** |
|  |  | Zero-order | .02 | .11 | **.27** | **.31** | .22 | **.22** | .25 | -.02 | -.17 | -.14 | -.10 | -.20 | **-.18** | -.13 | -.10 | -.19 | -.12 | **-.18** | **-.19** |
| 187 | Drank alcohol during working hours. | Partial | .04 | .01 | .11 | .10 | .01 | .04 | .07 | .03 | .01 | .01 | -.09 | **-.15** | -.06 | -.05 | .00 | -.02 | .01 | -.04 | -.07 |
|  |  | Zero-order | .02 | .00 | .07 | .04 | -.03 | .06 | .07 | .02 | .03 | .06 | -.07 | **-.11** | -.04 | -.02 | .02 | -.02 | -.03 | -.07 | -.10 |
| *Physical activity* | | | | | | | | | | | | | | | | | | | | | |
| 6 | Went running or jogging. | Partial | .07 | .01 | .13 | .03 | .04 | .03 | .05 | -.08 | **-.14** | -.02 | .10 | .00 | -.09 | -.07 | .07 | -.08 | -.05 | -.09 | -.05 |
|  |  | Zero-order | .14 | .09 | .27 | .20 | .21 | .10 | .14 | -.11 | **-.28** | -.12 | .04 | -.14 | -.16 | -.14 | -.07 | -.16 | -.07 | -.11 | -.04 |
| 173 | Went boating or rafting. | Partial | .21 | .05 | .12 | .04 | .03 | .03 | -.03 | .00 | **-.14** | -.01 | -.04 | -.06 | -.07 | -.09 | -.02 | -.05 | .04 | .00 | .01 |
|  |  | Zero-order | .23 | .08 | .17 | .10 | .10 | .08 | .02 | -.02 | **-.20** | -.04 | -.06 | -.11 | -.09 | -.11 | -.08 | -.09 | .00 | -.04 | -.01 |
| 196 | Participated in an exercise program. | Partial | .09 | .05 | .14 | .02 | .04 | .02 | -.01 | -.03 | -.08 | -.03 | .05 | -.01 | **-.16** | -.04 | .08 | -.06 | .01 | -.10 | -.04 |
|  |  | Zero-order | .15 | .12 | .27 | .18 | .20 | .13 | .11 | -.07 | -.24 | -.11 | .00 | -.15 | **-.21** | -.08 | -.07 | -.15 | -.06 | -.15 | -.07 |
| *Self-development and learning* | | | | | | | | | | | | | | | | | | | | | |
| 7 | Attended a public lecture. | Partial | **.24** | -.03 | .08 | -.11 | .03 | .00 | -.02 | -.10 | **-.14** | .05 | .11 | -.03 | .01 | -.07 | .03 | .01 | .00 | -.07 | -.07 |
|  |  | Zero-order | **.26** | .00 | .13 | -.04 | .09 | .01 | .00 | -.10 | **-.18** | .00 | .08 | -.07 | -.01 | -.09 | -.02 | -.02 | .01 | -.06 | -.06 |
| 40 | Visited an art exhibition. | Partial | **.28** | .07 | .06 | .00 | .00 | .03 | -.06 | -.07 | **-.14** | -.03 | -.06 | -.06 | -.07 | -.10 | .10 | -.05 | .08 | .02 | .02 |
|  |  | Zero-order | **.28** | .08 | .08 | .02 | .03 | .02 | -.06 | -.07 | **-.15** | -.05 | -.06 | -.07 | -.07 | -.11 | .08 | -.06 | .09 | .03 | .03 |
| 72 | Visited a museum. | Partial | .22 | .03 | .00 | -.08 | -.07 | -.04 | -.12 | -.01 | -.12 | .04 | .05 | .01 | -.02 | -.08 | .12 | .02 | **.12** | .01 | -.01 |
|  |  | Zero-order | .23 | .04 | .03 | -.05 | -.03 | -.04 | -.11 | -.01 | -.13 | .02 | .04 | -.02 | -.03 | -.09 | .09 | .01 | **.12** | .01 | .00 |
| 101 | Looked something up in an encyclopedia. | Partial | .12 | .00 | .00 | -.11 | -.01 | -.04 | -.13 | .04 | -.10 | **.15** | .10 | .05 | -.01 | -.04 | -.01 | .06 | .03 | -.01 | .02 |
|  |  | Zero-order | .15 | .03 | .06 | -.03 | .07 | -.02 | -.09 | .03 | -.15 | **.10** | .08 | -.01 | -.04 | -.07 | -.06 | .02 | .02 | -.02 | .02 |
| 113 | Read a book. | Partial | **.30** | .09 | .02 | -.05 | -.01 | -.05 | -.11 | -.04 | -.05 | .04 | .05 | -.03 | -.09 | -.09 | .01 | .00 | .02 | .06 | .08 |
|  |  | Zero-order | **.30** | .09 | .05 | .00 | .03 | -.07 | -.10 | -.03 | -.06 | -.02 | .03 | -.05 | -.10 | -.12 | -.02 | .00 | .06 | .10 | .11 |
| 128 | Studied some subject. | Partial | **.29** | .07 | -.01 | -.07 | .05 | -.06 | -.08 | .00 | -.08 | .08 | .04 | .00 | -.11 | -.11 | -.01 | .05 | .05 | .04 | -.01 |
|  |  | Zero-order | **.31** | .09 | .04 | -.01 | .11 | -.05 | -.05 | -.01 | -.12 | .04 | .02 | -.05 | -.13 | -.13 | -.05 | .02 | .05 | .04 | .01 |
| 145 | Read poetry. | Partial | **.23** | .08 | -.01 | -.10 | -.07 | -.05 | **-.20** | -.08 | **-.20** | .06 | .02 | .04 | -.05 | -.05 | **.19** | .08 | .11 | .06 | .04 |
|  |  | Zero-order | **.24** | .09 | .02 | -.06 | -.03 | -.07 | **-.19** | -.08 | **-.20** | .02 | .01 | .01 | -.06 | -.07 | **.16** | .07 | .13 | .08 | .07 |
| 160 | Learned a new skill. | Partial | **.25** | .05 | .09 | -.02 | .09 | .00 | -.04 | -.03 | -.08 | -.01 | -.05 | -.04 | -.05 | -.07 | -.01 | .01 | .02 | .01 | -.07 |
|  |  | Zero-order | **.29** | .10 | .21 | .12 | .22 | .05 | .04 | -.06 | -.20 | -.10 | -.09 | -.15 | -.10 | -.13 | -.11 | -.06 | .00 | -.02 | -.06 |
| 176 | Bought a book. | Partial | **.32** | .06 | -.04 | -.11 | -.06 | -.04 | -.11 | -.08 | -.03 | .05 | .05 | .01 | -.08 | -.09 | .03 | .04 | **.14** | .02 | .06 |
|  |  | Zero-order | **.30** | .04 | -.05 | -.11 | -.07 | -.07 | -.13 | -.07 | .00 | .04 | .05 | .02 | -.07 | -.10 | .04 | .06 | **.17** | .06 | .09 |
| 197 | Read a book about some artistic topic. | Partial | **.29** | .06 | .13 | -.04 | .01 | .00 | -.09 | -.10 | -.09 | .00 | -.02 | -.06 | -.09 | -.12 | .11 | -.04 | .09 | -.01 | -.01 |
|  |  | Zero-order | **.29** | .06 | .12 | -.03 | .01 | -.02 | -.10 | -.09 | -.08 | -.01 | -.03 | -.05 | -.08 | -.12 | .11 | -.03 | .10 | .01 | .00 |
| *Creative hobby* | | | | | | | | | | | | | | | | | | | | | |
| 50 | Produced a work of art. | Partial | .20 | .06 | .06 | -.03 | .06 | .02 | -.08 | -.05 | -.04 | .01 | -.05 | -.04 | -.08 | -.10 | **.13** | -.04 | -.03 | .04 | -.02 |
|  |  | Zero-order | .22 | .08 | .09 | .01 | .10 | .04 | -.05 | -.06 | -.08 | -.02 | -.06 | -.07 | -.09 | -.12 | **.09** | -.06 | -.03 | .04 | -.01 |
| 82 | Wrote poetry. | Partial | **.24** | .08 | .04 | -.04 | -.03 | .00 | -.12 | -.08 | **-.14** | -.03 | -.04 | -.04 | -.07 | -.06 | **.14** | .02 | .03 | **.12** | .06 |
|  |  | Zero-order | **.26** | .11 | .10 | .04 | .04 | .02 | -.08 | -.09 | **-.19** | -.07 | -.06 | -.09 | -.10 | -.09 | **.08** | -.01 | .03 | **.10** | .07 |
| 178 | Played a musical instrument. | Partial | **.24** | .04 | .08 | -.08 | -.01 | -.03 | -.14 | -.08 | -.12 | .02 | .04 | -.02 | -.01 | -.10 | .05 | .01 | .06 | .07 | .05 |
|  |  | Zero-order | **.27** | .08 | .16 | .02 | .08 | .03 | -.07 | -.10 | -.20 | -.03 | .02 | -.09 | -.04 | -.12 | -.02 | -.04 | .02 | .03 | .03 |
| 200 | Sang or played an instrument in public. | Partial | .16 | .03 | .02 | -.06 | -.08 | .01 | **-.18** | -.11 | **-.17** | .03 | .11 | .03 | -.02 | .00 | .02 | .03 | .08 | .09 | .08 |
|  |  | Zero-order | .19 | .06 | .11 | .04 | .02 | .05 | **-.12** | -.12 | **-.24** | -.03 | .08 | -.04 | -.06 | -.04 | -.05 | -.02 | .06 | .07 | .08 |
| *Playing games* | | | | | | | | | | | | | | | | | | | | | |
| 54 | Worked on a jigsaw puzzle. | Partial | -.03 | -.08 | -.03 | -.05 | -.13 | -.02 | -.05 | -.02 | .04 | .06 | .10 | **.14** | .04 | .02 | .05 | .03 | .00 | -.03 | -.06 |
|  |  | Zero-order | -.02 | -.08 | -.01 | -.01 | -.09 | -.04 | -.06 | -.01 | .03 | .02 | .08 | **.11** | .03 | .00 | .03 | .03 | .03 | .00 | -.03 |
| 69 | Gambled with cards or dice. | Partial | -.02 | -.05 | .11 | .09 | .03 | .07 | .08 | .00 | -.04 | -.04 | -.03 | **-.16** | -.01 | .00 | .00 | -.09 | .00 | .00 | -.07 |
|  |  | Zero-order | .02 | .00 | .17 | .15 | .10 | .14 | .14 | -.03 | -.12 | -.05 | -.04 | **-.20** | -.04 | .00 | -.06 | -.14 | -.06 | -.06 | -.11 |
| 134 | Gambled on a slot machine or video poker game. | Partial | -.05 | -.07 | .15 | .14 | .10 | .10 | .13 | -.01 | -.06 | -.11 | -.08 | -.10 | .03 | -.10 | -.01 | **-.13** | .03 | -.05 | -.06 |
|  |  | Zero-order | -.01 | -.01 | .21 | .20 | .16 | .18 | .19 | -.05 | -.15 | -.11 | -.09 | -.16 | -.01 | -.10 | -.08 | **-.19** | -.04 | -.12 | -.11 |
| 166 | Went to a casino. | Partial | -.04 | -.06 | .10 | .10 | .02 | .12 | .13 | .01 | .00 | -.07 | -.06 | -.07 | .03 | -.06 | .00 | -.05 | -.05 | **-.12** | **-.11** |
|  |  | Zero-order | -.04 | -.04 | .09 | .09 | .01 | .17 | .15 | -.01 | -.02 | -.04 | -.05 | -.06 | .03 | -.04 | -.01 | -.07 | -.10 | **-.16** | **-.16** |
| 198 | Bet money on a sports event. | Partial | -.03 | -.01 | **.17** | .11 | .03 | .08 | .08 | -.05 | .00 | -.06 | -.02 | -.06 | -.04 | -.03 | -.04 | -.11 | -.01 | -.06 | -.08 |
|  |  | Zero-order | .01 | .05 | **.23** | .18 | .11 | .17 | .16 | -.09 | -.11 | -.06 | -.04 | -.12 | -.08 | -.03 | -.11 | -.17 | -.10 | -.14 | -.13 |
| *Hobby* | | | | | | | | | | | | | | | | | | | | | |
| 127 | Read a fashion-related book. | Partial | -.04 | **-.13** | .12 | .04 | .06 | .02 | .09 | -.02 | .02 | -.11 | .02 | .01 | .09 | -.05 | .05 | -.05 | -.01 | **-.15** | **-.15** |
|  |  | Zero-order | -.05 | **-.16** | .06 | .00 | .02 | -.06 | .02 | .01 | .08 | -.12 | .02 | .04 | .10 | -.06 | .08 | .00 | .07 | **-.06** | **-.08** |
| 194 | Went fishing or hunting. | Partial | .00 | -.06 | .03 | -.02 | -.06 | .00 | -.04 | -.05 | -.03 | .00 | .02 | .02 | -.01 | .02 | **.14** | .02 | -.01 | -.03 | -.02 |
|  |  | Zero-order | .02 | -.02 | .07 | .03 | -.01 | .08 | .02 | -.08 | -.09 | .01 | .01 | -.02 | -.03 | .02 | **.09** | -.02 | -.07 | -.09 | -.06 |
| *Music* | | | | | | | | | | | | | | | | | | | | | |
| 207 | Used an mp3 player or Ipod. | Partial | .21 | **.14** | .15 | .12 | .08 | .07 | .02 | -.03 | -.14 | -.08 | -.06 | -.07 | **-.15** | **-.20** | -.07 | -.07 | .11 | -.02 | .01 |
|  |  | Zero-order | .27 | **.22** | .33 | .32 | .29 | .15 | .14 | -.08 | -.32 | -.20 | -.12 | -.24 | **-.22** | **-.25** | -.22 | -.18 | .05 | -.06 | .01 |
| *Using internet (besides communication)* | | | | | | | | | | | | | | | | | | | | | |
| 60 | Surfed the Internet. | Partial | .07 | .07 | -.06 | -.03 | .06 | -.09 | -.03 | .07 | -.03 | .02 | .00 | -.01 | -.05 | -.03 | -.03 | .00 | .07 | **.10** | .05 |
|  |  | Zero-order | .15 | .14 | .12 | .17 | .25 | -.01 | .07 | .03 | -.20 | -.11 | -.06 | -.16 | -.12 | -.11 | -.16 | -.10 | .04 | **.06** | .05 |
| 65 | Downloaded music from the Internet. | Partial | .08 | .04 | **.18** | .13 | **.16** | .04 | .08 | -.01 | -.13 | -.12 | -.07 | -.13 | -.09 | -.08 | -.12 | -.06 | .04 | .02 | .02 |
|  |  | Zero-order | .19 | .16 | **.39** | .38 | **.40** | .16 | .22 | -.07 | -.37 | -.25 | -.14 | -.32 | -.19 | -.17 | -.30 | -.20 | -.03 | -.04 | .01 |
| 75 | Made an entry on a personal web-page. | Partial | .09 | .01 | .09 | .01 | .07 | .05 | .04 | .00 | -.06 | -.05 | -.02 | .00 | -.08 | **-.15** | .00 | -.07 | .04 | -.01 | .01 |
|  |  | Zero-order | .16 | .09 | .26 | .21 | .26 | .12 | .14 | -.04 | -.24 | -.16 | -.07 | -.16 | -.15 | **-.21** | -.15 | -.16 | .01 | -.04 | .02 |
| *Social interaction* | | | | | | | | | | | | | | | | | | | | | |
| 4 | Discussed sports. | Partial | .05 | .03 | .03 | .02 | -.04 | -.08 | -.03 | **-.11** | -.04 | .07 | .08 | .01 | -.06 | -.03 | .05 | .00 | .03 | .04 | .02 |
|  |  | Zero-order | .08 | .07 | .09 | .08 | .03 | .01 | .04 | **-.14** | -.12 | .06 | .07 | -.05 | -.08 | -.02 | -.01 | -.06 | -.05 | -.03 | -.03 |
| 10 | Used a computer for social networking. | Partial | .00 | -.04 | .14 | .13 | .02 | .10 | .12 | -.02 | -.02 | -.12 | -.10 | -.06 | .00 | -.10 | .00 | -.02 | .06 | **-.12** | **-.14** |
|  |  | Zero-order | .07 | .03 | .26 | .26 | .18 | .14 | .18 | -.04 | -.16 | -.21 | -.14 | -.18 | -.07 | -.15 | -.12 | -.10 | .05 | **-.13** | **-.12** |
| 97 | Traded music with a friend. | Partial | .06 | .04 | **.19** | .17 | .10 | .06 | .05 | -.08 | **-.16** | -.13 | -.07 | **-.13** | -.05 | -.13 | -.04 | -.06 | .10 | -.01 | .02 |
|  |  | Zero-order | .14 | .12 | **.34** | .34 | .29 | .12 | .15 | -.10 | **-.32** | -.24 | -.13 | **-.27** | -.13 | -.20 | -.18 | -.15 | .07 | -.03 | .03 |
| 108 | Participated in an online discussion group. | Partial | .13 | -.02 | .16 | .07 | .06 | .08 | .02 | -.05 | -.11 | -.02 | -.02 | -.08 | -.09 | **-.15** | -.05 | -.04 | .11 | -.03 | -.01 |
|  |  | Zero-order | .20 | .07 | .30 | .24 | .23 | .15 | .13 | -.09 | -.27 | -.12 | -.07 | -.22 | -.16 | **-.20** | -.18 | -.13 | .05 | -.07 | -.02 |
| *Dating & Partying* | | | | | | | | | | | | | | | | | | | | | |
| 53 | Went on a date. | Partial | .04 | .05 | .09 | .06 | .07 | .07 | .08 | .03 | .01 | -.06 | -.02 | -.03 | -.07 | **-.15** | -.09 | -.06 | -.01 | -.04 | -.03 |
|  |  | Zero-order | .12 | .12 | .24 | .23 | .24 | .13 | .17 | -.01 | -.16 | -.17 | -.07 | -.17 | -.14 | **-.20** | -.21 | -.15 | -.03 | -.06 | -.02 |
| 78 | Went to a large party. | Partial | .02 | .07 | **.17** | .17 | .12 | .09 | .15 | -.03 | -.09 | -.08 | -.02 | **-.16** | -.13 | **-.14** | -.08 | -.11 | .00 | -.04 | -.02 |
|  |  | Zero-order | .09 | .12 | **.28** | .30 | .26 | .13 | .22 | -.05 | -.22 | -.17 | -.06 | **-.26** | -.19 | **-.19** | -.18 | -.17 | -.01 | -.05 | .00 |
| 202 | Had a blind date. | Partial | .01 | .01 | .09 | .11 | -.01 | .04 | .08 | .02 | -.07 | **-.14** | -.08 | -.10 | .01 | -.04 | -.04 | .01 | **.12** | -.04 | -.03 |
|  |  | Zero-order | .03 | .03 | .13 | .16 | .05 | .05 | .10 | .01 | -.12 | **-.17** | -.09 | -.14 | -.02 | -.06 | -.08 | -.02 | **.11** | -.04 | -.02 |
| *Transportation* | | | | | | | | | | | | | | | | | | | | | |
| 22 | Used public transportation. | Partial | .11 | .01 | -.02 | -.03 | -.07 | -.09 | -.11 | .00 | -.01 | .04 | .02 | -.06 | -.03 | .01 | .05 | .07 | .07 | **.11** | .12 |
|  |  | Zero-order | .16 | .06 | .11 | .12 | .09 | -.06 | -.04 | -.02 | -.13 | -.08 | -.03 | -.16 | -.08 | -.06 | -.06 | .00 | .08 | **.11** | .15 |
| 59 | Rode a motorcycle. | Partial | -.08 | -.03 | .12 | .03 | .07 | .10 | .11 | .03 | -.06 | -.06 | .00 | -.02 | -.04 | -.05 | .02 | -.06 | -.08 | -.04 | **-.12** |
|  |  | Zero-order | -.04 | .01 | .18 | .10 | .13 | .16 | .16 | -.01 | -.14 | -.07 | -.01 | -.08 | -.07 | -.05 | -.05 | -.11 | -.13 | -.09 | **-.16** |
| 138 | Traveled by train or plane. | Partial | .17 | .01 | .03 | -.07 | -.01 | .00 | -.06 | **-.12** | -.08 | .00 | .05 | .02 | -.04 | -.05 | .03 | -.01 | .07 | .02 | .08 |
|  |  | Zero-order | .20 | .06 | .12 | .05 | .09 | .03 | .00 | **-.13** | -.17 | -.07 | .02 | -.06 | -.09 | -.09 | -.05 | -.06 | .06 | .01 | .08 |
| *Religious practices* | | | | | | | | | | | | | | | | | | | | | |
| 2 | Discussed religion or spirituality. | Partial | .12 | -.03 | -.13 | -.16 | **-.17** | -.11 | -.17 | **-.13** | -.02 | **.17** | **.19** | .09 | .05 | .02 | .10 | .12 | .03 | .05 | .08 |
|  |  | Zero-order | .11 | -.03 | -.14 | -.17 | **-.18** | -.11 | -.17 | **-.13** | .01 | **.18** | **.20** | .10 | .06 | .03 | .11 | .13 | .03 | .05 | .08 |
| 33 | Prayed (not including blessings at meals). | Partial | -.05 | **-.14** | **-.16** | **-.22** | -.09 | -.10 | -.13 | -.08 | .04 | **.14** | **.34** | **.13** | .13 | .08 | .02 | **.13** | -.03 | .01 | .03 |
|  |  | Zero-order | -.09 | **-.18** | **-.23** | **-.29** | -.17 | -.15 | -.18 | -.05 | .13 | **.17** | **.35** | **.19** | .16 | .10 | .09 | **.18** | .01 | .05 | .05 |
| 66 | Read the Bible or other sacred text. | Partial | .10 | -.05 | **-.16** | **-.25** | **-.17** | -.10 | **-.28** | -.09 | -.07 | **.17** | **.32** | .10 | .10 | .07 | .07 | **.18** | .01 | .09 | .10 |
|  |  | Zero-order | .09 | -.06 | **-.16** | **-.24** | **-.17** | -.11 | **-.28** | -.09 | -.05 | **.17** | **.32** | .11 | .11 | .07 | .08 | **.18** | .03 | .10 | .11 |
| 98 | Gave a blessing at a meal. | Partial | .04 | **-.17** | -.14 | **-.20** | **-.16** | -.10 | **-.23** | **-.14** | -.09 | **.14** | **.36** | .13 | **.18** | .11 | .04 | **.18** | -.01 | .07 | .08 |
|  |  | Zero-order | .01 | **-.19** | -.18 | **-.24** | **-.20** | -.12 | **-.25** | **-.13** | -.02 | **.17** | **.37** | .17 | **.20** | .13 | .08 | **.20** | -.01 | .07 | .08 |
| 131 | Attended a church or religious service. | Partial | -.04 | **-.12** | -.13 | **-.20** | -.08 | -.08 | -.16 | -.05 | -.03 | **.15** | **.33** | .11 | .08 | .10 | -.04 | **.12** | -.03 | .06 | .08 |
|  |  | Zero-order | -.07 | **-.15** | -.18 | **-.24** | -.14 | -.13 | -.20 | -.02 | .05 | **.17** | **.34** | .15 | .11 | .12 | .01 | **.16** | .01 | .09 | .09 |
| 163 | Listened to a religious program on the radio or TV. | Partial | .07 | **-.17** | -.13 | **-.25** | **-.21** | -.04 | **-.23** | **-.13** | -.10 | **.19** | **.37** | .10 | **.16** | **.15** | .08 | **.13** | .01 | -.03 | .01 |
|  |  | Zero-order | .01 | **-.21** | -.23 | **-.34** | **-.31** | -.10 | **-.28** | **-.09** | .04 | **.24** | **.39** | .19 | **.21** | **.19** | .17 | **.19** | .03 | .00 | .01 |
| 193 | Read a book about religion or spirituality. | Partial | .18 | .01 | -.12 | **-.24** | **-.15** | -.01 | **-.22** | **-.17** | -.08 | **.14** | **.27** | .05 | .07 | .04 | .08 | .11 | .03 | .00 | .04 |
|  |  | Zero-order | .14 | -.02 | -.17 | **-.28** | **-.20** | -.05 | **-.25** | **-.14** | .00 | **.17** | **.29** | .11 | .09 | .06 | .13 | .14 | .05 | .02 | .05 |
| *Supporting others* | | | | | | | | | | | | | | | | | | | | | |
| 83 | Donated money to charity. | Partial | .06 | -.07 | -.07 | -.12 | -.10 | -.02 | -.06 | -.05 | -.06 | **.13** | .13 | .07 | -.02 | .02 | .10 | .05 | -.01 | -.01 | -.01 |
|  |  | Zero-order | .01 | -.12 | -.17 | -.22 | -.20 | -.07 | -.12 | -.03 | .06 | **.19** | .16 | .16 | .03 | .06 | .18 | .11 | .02 | .01 | -.01 |
| 144 | Volunteered for a club or organization. | Partial | .09 | -.10 | -.01 | -.13 | -.08 | .01 | -.10 | -.10 | **-.14** | -.01 | .08 | .03 | .05 | .04 | .11 | .07 | .09 | .04 | .01 |
|  |  | Zero-order | .12 | -.07 | .05 | -.04 | .00 | .01 | -.07 | -.10 | **-.18** | -.07 | .05 | -.02 | .02 | -.01 | .05 | .05 | .10 | .06 | .04 |
| *Environmental Activities* | | | | | | | | | | | | | | | | | | | | | |
| 55 | Composted food scraps or yard waste. | Partial | .10 | -.03 | -.07 | -.12 | **-.15** | -.11 | -.17 | -.07 | -.03 | .04 | .06 | .10 | .05 | .06 | **.21** | .00 | .11 | .07 | .02 |
|  |  | Zero-order | .04 | -.08 | -.16 | -.22 | **-.24** | -.14 | -.21 | -.05 | .07 | .11 | .09 | .18 | .09 | .10 | **.28** | .06 | .11 | .08 | .01 |
| 120 | Changed a habit to have less impact on the environment. | Partial | .06 | -.10 | -.01 | -.08 | -.08 | -.05 | -.09 | -.10 | .04 | .07 | .07 | .06 | -.03 | -.01 | **.25** | .04 | .04 | -.05 | -.07 |
|  |  | Zero-order | .03 | -.13 | -.06 | -.13 | -.13 | -.09 | -.13 | -.09 | .10 | .09 | .08 | .11 | .00 | .00 | **.28** | .08 | .06 | -.02 | -.05 |
| 152 | Used both sides of a piece of paper before discarding it. | Partial | .13 | -.01 | -.10 | -.16 | -.04 | -.11 | **-.18** | .01 | .00 | .13 | .07 | .06 | -.02 | .00 | .10 | .07 | .08 | .09 | .03 |
|  |  | Zero-order | .15 | .01 | -.04 | -.09 | .02 | -.09 | **-.14** | .00 | -.05 | .09 | .05 | .02 | -.04 | -.02 | .06 | .05 | .08 | .09 | .04 |
| 192 | Picked up litter. | Partial | .06 | -.05 | -.01 | -.08 | -.09 | -.08 | **-.18** | -.09 | -.05 | .06 | .07 | .06 | .04 | .02 | .10 | **.13** | **.12** | .05 | .01 |
|  |  | Zero-order | .03 | -.08 | -.07 | -.14 | -.15 | -.11 | **-.21** | -.08 | .02 | .10 | .09 | .11 | .07 | .04 | .15 | **.16** | **.12** | .06 | .01 |
| *Finance/Investment* | | | | | | | | | | | | | | | | | | | | | |
| 16 | Obtained stock market prices. | Partial | .09 | -.03 | .12 | .03 | .00 | **.13** | .06 | .00 | .01 | -.07 | -.07 | -.03 | -.08 | .00 | -.01 | -.06 | -.01 | **-.10** | -.10 |
|  |  | Zero-order | .08 | -.03 | .09 | .00 | -.02 | **.14** | .07 | -.01 | .02 | -.03 | -.06 | -.01 | -.07 | .02 | .01 | -.06 | -.04 | **-.13** | -.13 |
| 56 | Read a book on a financial topic. | Partial | .11 | -.02 | .08 | -.05 | .04 | .08 | .05 | -.05 | -.02 | -.06 | .03 | .05 | -.07 | -.04 | .03 | -.09 | .01 | -.10 | **-.14** |
|  |  | Zero-order | .09 | -.04 | .04 | -.09 | -.01 | .07 | .03 | -.04 | .01 | -.02 | .04 | .08 | -.05 | -.02 | .06 | -.07 | .00 | -.10 | **-.15** |
| 81 | Bought or sold stocks or bonds. | Partial | .09 | -.07 | .05 | -.05 | .00 | .12 | .05 | .03 | .03 | -.01 | -.03 | -.02 | -.06 | -.01 | .03 | -.06 | .00 | -.10 | **-.15** |
|  |  | Zero-order | .05 | -.09 | -.02 | -.12 | -.08 | .12 | .03 | .03 | .08 | .06 | -.01 | .04 | -.02 | .03 | .08 | -.03 | -.03 | -.12 | **-.18** |
| 116 | Donated money to a political campaign or cause. | Partial | .02 | **-.16** | .10 | -.04 | -.03 | .11 | .09 | .03 | .01 | -.05 | .03 | -.04 | .00 | .00 | .05 | -.02 | -.04 | **-.10** | **-.15** |
|  |  | Zero-order | .00 | **-.17** | .05 | -.08 | -.07 | .11 | .07 | .03 | .04 | -.01 | .05 | -.01 | .02 | .02 | .08 | .00 | -.05 | **-.11** | **-.17** |
| 146 | Purchased a commodity as an investment. | Partial | .09 | -.03 | .04 | -.04 | .01 | .06 | .04 | -.01 | .04 | .02 | .01 | -.03 | -.06 | -.04 | .07 | -.07 | -.01 | -.06 | **-.15** |
|  |  | Zero-order | .05 | -.05 | -.04 | -.12 | -.08 | .07 | .02 | -.01 | .09 | .09 | .04 | .04 | -.02 | .01 | .12 | -.04 | -.04 | -.09 | **-.18** |
| *Other* | | | | | | | | | | | | | | | | | | | | | |
| 12 | Made an entry in a diary or journal. | Partial | .22 | .01 | .02 | -.04 | .00 | -.02 | -.06 | -.03 | -.06 | .01 | -.04 | -.01 | -.05 | -.05 | .01 | .06 | **.12** | .00 | -.02 |
|  |  | Zero-order | .22 | .01 | .03 | -.01 | .03 | -.06 | -.07 | -.02 | -.06 | -.03 | -.05 | -.03 | -.05 | -.08 | .00 | .06 | **.16** | .05 | .03 |
| 17 | Tried something completely new. | Partial | .16 | .05 | **.17** | .09 | .07 | .05 | -.01 | .01 | -.09 | -.05 | -.11 | -.07 | -.10 | -.08 | .03 | -.07 | .02 | -.03 | -.06 |
|  |  | Zero-order | .21 | .11 | **.28** | .21 | .20 | .11 | .07 | -.02 | -.21 | -.13 | -.15 | -.17 | -.16 | -.13 | -.08 | -.14 | -.01 | -.06 | -.06 |
| 95 | Bought a self-help book. | Partial | .01 | **-.11** | .08 | .05 | .05 | .02 | .08 | .01 | .00 | -.04 | .03 | .03 | .01 | -.01 | .01 | -.08 | -.06 | **-.10** | -.12 |
|  |  | Zero-order | -.04 | **-.15** | -.02 | -.05 | -.05 | -.05 | .01 | .04 | .10 | .00 | .05 | .10 | .05 | .01 | .08 | -.02 | -.01 | **-.04** | -.08 |
| 110 | Wrote a thank-you note. | Partial | .15 | -.09 | .00 | -.08 | -.08 | -.01 | -.13 | -.11 | -.05 | .00 | **.15** | .03 | .03 | .04 | .05 | .03 | .07 | .00 | .02 |
|  |  | Zero-order | .16 | -.08 | .03 | -.04 | -.04 | .00 | -.11 | -.11 | -.08 | -.02 | **.14** | .00 | .01 | .03 | .02 | .01 | .06 | .00 | .02 |
| 174 | Worked on a scrap book. | Partial | .06 | -.04 | .01 | -.01 | -.07 | -.02 | -.07 | .00 | -.05 | -.05 | **.18** | .03 | .04 | -.05 | -.03 | -.02 | .03 | .01 | .06 |
|  |  | Zero-order | .06 | -.05 | .00 | -.01 | -.07 | -.06 | -.09 | .02 | -.02 | -.07 | **.17** | .03 | .04 | -.06 | -.02 | .00 | .07 | .05 | .10 |
| 177 | Worked on a retirement plan. | Partial | .04 | -.05 | -.04 | -.09 | -.03 | .08 | .00 | -.02 | .07 | -.01 | .04 | **.14** | -.06 | .04 | .02 | -.08 | -.06 | -.05 | -.04 |
|  |  | Zero-order | -.04 | -.12 | -.19 | -.25 | -.20 | .02 | -.09 | .01 | .22 | .11 | .09 | **.26** | .02 | .11 | .14 | .02 | -.04 | -.03 | -.06 |
| 180 | Participated in an animal show. | Partial | -.01 | -.09 | .05 | -.02 | -.02 | .03 | .00 | -.05 | .01 | -.01 | .03 | -.02 | .01 | .02 | **.13** | -.02 | -.02 | -.08 | -.08 |
|  |  | Zero-order | -.04 | -.11 | -.01 | -.08 | -.09 | .01 | -.04 | -.04 | .07 | .04 | .05 | .04 | .04 | .05 | **.18** | .02 | -.01 | -.06 | -.08 |
| 188 | Attended a town meeting. | Partial | .02 | -.09 | .02 | .00 | .00 | **.13** | .03 | -.05 | -.01 | -.01 | .04 | -.03 | -.02 | -.03 | .02 | -.03 | .02 | -.08 | -.09 |
|  |  | Zero-order | -.02 | -.11 | -.06 | -.09 | -.09 | **.11** | .00 | -.04 | .06 | .06 | .07 | .04 | .02 | .02 | .08 | .01 | .01 | -.09 | -.11 |
| 10 highest correlates aggregated | | Partial | .45 | .29 | .33 | .37 | .34 | .32 | .37 | .25 | .28 | .27 | .45 | .30 | .30 | .30 | .34 | .27 | .25 | .31 | .32 |
|  |  | Zero-order | .47 | .34 | .46 | .44 | .47 | .40 | .41 | .23 | .39 | .29 | .45 | .40 | .34 | .33 | .40 | .33 | .28 | .30 | .34 |

*Note.* SDT = self-direction-thought; SDA = self-direction-action; ST = stimulation; HE = hedonism; AC = achievement; POD = power-dominance; POR = power-resources; FAC = face; SEP = security-personal; SES = security-societal; TR = tradition; COR = conformity-rules; COI = conformity-interpersonal; HU = humility; UNN = universalism-nature; UNC = universalism-concern; UNT = universalism-tolerance; BED = benevolence-dependability; BEC = benevolence-caring.

The 10 highest correlates for each value are in bold. To calculate aggregate correlations we reversed the highest correlates with a negative sign.

*N* = 703. All coefficients > |.07| are significant at *p* < .05. All coefficients > |.10| are significant at *p* < .01. All coefficients > |.12| are significant at *p* < .001.

Table B

*Correlations between raw value traits (not centered) and 88 behavioral acts*

| No | Item | Correlation | SDT | SDA | ST | HE | AC | POD | POR | FAC | SEP | SES | TR | COR | COI | HU | UNN | UNC | UNT | BEC | BED |
| --- | --- | --- | --- | --- | --- | --- | --- | --- | --- | --- | --- | --- | --- | --- | --- | --- | --- | --- | --- | --- | --- |
| *Homemaking* | | | | | | | | | | | | | | | | | | | | | |
| 5* | Washed dishes. | partial | .10 | .04 | -.04 | -.07 | -.01 | -.10 | -.09 | .02 | .04 | .11 | .13 | .09 | .08 | .08 | .05 | **.15** | **.16** | .10 | **.13** |
|  |  | zero-order | .09 | .03 | -.08 | -.11 | -.04 | -.14 | -.12 | .05 | .11 | .13 | .15 | .14 | .11 | .10 | .11 | **.20** | **.20** | .14 | **.17** |
| 9* | Cared for a potted plant. | partial | .11 | .00 | -.02 | -.01 | -.03 | -.12 | -.08 | -.01 | .02 | .06 | .04 | .08 | .09 | .04 | **.17** | .10 | .10 | **.13** | .09 |
|  |  | zero-order | .07 | -.04 | -.11 | -.11 | -.12 | -.21 | -.16 | .06 | .18 | .11 | .09 | .19 | .16 | .09 | **.27** | .19 | .19 | **.22** | .15 |
| 38* | Made a bed. | partial | .05 | .01 | -.01 | -.03 | .03 | **-.14** | -.09 | .06 | .01 | .05 | .08 | .06 | .08 | .07 | .03 | .11 | **.14** | .10 | .06 |
|  |  | zero-order | .06 | .01 | .00 | -.01 | .03 | **-.15** | -.09 | .07 | .02 | .04 | .08 | .06 | .08 | .07 | .04 | .12 | **.16** | .12 | .08 |
| 70* | Cleaned the house. | partial | .15 | .05 | .08 | .01 | .01 | -.10 | -.07 | .01 | .07 | .09 | .14 | .08 | .09 | **.13** | .11 | **.15** | **.21** | **.16** | **.15** |
|  |  | zero-order | .14 | .05 | .04 | -.01 | -.01 | -.16 | -.11 | .07 | .15 | .10 | .16 | .13 | .12 | **.13** | .16 | **.20** | **.27** | **.24** | **.21** |
| 103* | Ironed linens or clothes. | partial | .14 | .03 | .02 | -.02 | .06 | -.10 | -.02 | .02 | .09 | **.18** | **.24** | **.16** | **.13** | **.14** | .09 | **.19** | **.17** | **.16** | **.19** |
|  |  | zero-order | .13 | .02 | -.02 | -.04 | .03 | -.17 | -.08 | .08 | .18 | **.18** | **.25** | **.21** | **.17** | **.14** | .15 | **.25** | **.26** | **.25** | **.25** |
| 135* | Cooked a meal. | partial | .10 | .06 | -.04 | -.03 | -.01 | -.10 | -.10 | .01 | .04 | .10 | .12 | .11 | .03 | .09 | .08 | .10 | **.12** | .08 | .10 |
|  |  | zero-order | .10 | .05 | -.05 | -.04 | -.02 | -.16 | -.13 | .06 | .11 | .10 | .14 | .15 | .07 | .09 | .12 | .16 | **.19** | .16 | .17 |
| 167* | Baked a cake, pie, cookies, or bread. | partial | .07 | -.01 | .02 | -.01 | .03 | -.05 | .00 | -.03 | .04 | .08 | .12 | .11 | .10 | .09 | .11 | .09 | .10 | **.12** | .11 |
|  |  | zero-order | .07 | -.01 | -.01 | -.02 | .01 | -.15 | -.07 | .06 | .16 | .08 | .14 | .17 | .14 | .09 | .18 | .18 | .22 | **.25** | .22 |
| *Gardening* | | | | | | | | | | | | | | | | | | | | | |
| 42* | Gardened. | partial | .01 | -.04 | .04 | -.01 | .00 | -.01 | -.02 | -.02 | .02 | .05 | .12 | .08 | .02 | .07 | **.17** | .05 | .04 | .08 | .04 |
|  |  | zero-order | -.03 | -.07 | -.05 | -.11 | -.09 | -.04 | -.07 | -.01 | .09 | .10 | .14 | .15 | .06 | .11 | **.23** | .08 | .03 | .08 | .02 |
| 107* | Planted or transplanted a plant. | partial | .01 | -.05 | -.02 | -.08 | -.02 | -.03 | -.03 | .00 | .07 | .03 | .08 | .07 | .10 | .08 | **.16** | .05 | .04 | .06 | .03 |
|  |  | zero-order | -.05 | -.11 | -.17 | -.23 | -.17 | -.11 | -.13 | .05 | .22 | .13 | .13 | .20 | .16 | .14 | **.27** | .14 | .08 | .10 | .05 |
| *Shopping* | | | | | | | | | | | | | | | | | | | | | |
| 61 | Spent an hour or more in a non-grocery store. | partial | .11 | **.12** | .10 | .13 | **.15** | .05 | .12 | .05 | .06 | .02 | .03 | .00 | .01 | -.04 | .07 | .03 | .07 | .03 | .04 |
|  |  | zero-order | .13 | **.13** | .12 | .16 | **.18** | .03 | .11 | .07 | .06 | .00 | .03 | -.01 | .01 | -.06 | .06 | .04 | .11 | .08 | .09 |
| 126 | Checked the sales ads in a newspaper. | partial | .04 | .04 | .06 | .09 | **.14** | .00 | .13 | .08 | **.15** | .05 | .09 | .01 | -.01 | .00 | .06 | .01 | .02 | .06 | .04 |
|  |  | zero-order | .05 | .04 | .06 | .09 | **.14** | .00 | .13 | .08 | **.14** | .04 | .09 | .01 | -.01 | .00 | .05 | .01 | .03 | .07 | .04 |
| 159 | Bought a fashionable item of clothing. | partial | .10 | .06 | .16 | .16 | **.17** | .07 | **.20** | .09 | .09 | .03 | .05 | .05 | .02 | -.03 | .06 | .01 | .06 | .05 | .04 |
|  |  | zero-order | .15 | .10 | .23 | .25 | **.25** | .05 | **.20** | .11 | .04 | -.03 | .03 | -.01 | .00 | -.08 | .01 | .01 | .12 | .11 | .10 |
| 162* | Shopped in a music store. | partial | .14 | .01 | .10 | .00 | -.04 | -.02 | -.08 | **-.11** | -.10 | -.06 | -.03 | -.03 | -.03 | -.07 | .03 | .01 | .09 | .02 | .00 |
|  |  | zero-order | .14 | .02 | .12 | .04 | -.01 | .00 | -.05 | **-.12** | -.13 | -.08 | -.04 | -.06 | -.05 | -.08 | .00 | -.02 | .07 | .01 | -.01 |
| 179* | Shopped on the web. | partial | .16 | **.10** | .06 | .01 | .11 | .02 | .07 | .00 | -.04 | .03 | .03 | .01 | -.10 | -.09 | .00 | .00 | .06 | .05 | .04 |
|  |  | zero-order | .18 | **.13** | .14 | .11 | .19 | .08 | .13 | -.04 | -.15 | -.03 | -.01 | -.09 | -.15 | -.13 | -.09 | -.06 | .01 | -.01 | .01 |
| 189* | Examined the clothing categories on eBay. | partial | .08 | .09 | .16 | .14 | **.19** | .03 | .16 | **.14** | .04 | .07 | .05 | -.03 | -.04 | -.07 | .02 | -.01 | .05 | .03 | .02 |
|  |  | zero-order | .13 | .14 | .25 | .25 | **.28** | .03 | .19 | **.13** | -.03 | -.01 | .02 | -.10 | -.06 | -.12 | -.04 | -.03 | .09 | .07 | .07 |
| *Being alone* | | | | | | | | | | | | | | | | | | | | | |
| 23 | Ate dinner alone. | partial | .11 | .10 | .08 | -.01 | .06 | -.02 | -.05 | .04 | -.06 | .07 | .05 | .00 | -.01 | .02 | -.01 | .09 | .08 | .08 | **.13** |
|  |  | zero-order | .14 | .13 | .16 | .08 | .14 | .02 | .01 | .02 | -.14 | .01 | .02 | -.08 | -.05 | -.02 | -.09 | .04 | .05 | .05 | **.11** |
| 49* | Went to the movies alone. | partial | .09 | .04 | .07 | .01 | -.05 | .02 | -.04 | **-.12** | .00 | -.04 | -.05 | -.01 | .01 | .02 | .01 | -.01 | .08 | -.01 | .03 |
|  |  | zero-order | .07 | .03 | .03 | -.04 | -.08 | .00 | -.06 | **-.11** | .03 | -.01 | -.04 | .02 | .03 | .04 | .04 | .01 | .08 | .00 | .03 |
| 88* | Went to a concert or theater alone. | partial | .16 | -.02 | .10 | -.04 | -.11 | .02 | -.06 | **-.10** | -.08 | -.04 | .00 | -.01 | .02 | .00 | .03 | .01 | .07 | -.02 | -.02 |
|  |  | zero-order | .13 | -.04 | .05 | -.09 | -.15 | .01 | -.09 | **-.09** | -.04 | -.01 | .01 | .03 | .04 | .02 | .06 | .03 | .07 | -.01 | -.03 |
| 121 | Chose to spend a day by myself. | partial | .16 | **.14** | .10 | .06 | .05 | .06 | .02 | .09 | .00 | .02 | -.01 | -.01 | -.04 | -.03 | .05 | .02 | .10 | .04 | .04 |
|  |  | zero-order | .19 | **.17** | .16 | .14 | .12 | .08 | .06 | .08 | -.06 | -.03 | -.03 | -.07 | -.06 | -.07 | -.01 | -.01 | .10 | .04 | .05 |
| *Drinking alcohol* | | | | | | | | | | | | | | | | | | | | | |
| 62* | Drank whiskey, vodka, gin, or other hard liquor. | partial | .01 | .06 | .13 | **.19** | **.15** | .12 | **.21** | .07 | .03 | -.04 | -.01 | -.10 | -.08 | -.07 | -.04 | -.06 | .01 | .03 | .00 |
|  |  | zero-order | .00 | .05 | .14 | **.18** | **.14** | .16 | **.23** | .03 | -.04 | -.05 | -.03 | -.13 | -.10 | -.07 | -.08 | -.10 | -.06 | -.05 | -.06 |
| 92* | Drank in a bar or night club. | partial | .05 | .08 | **.16** | **.20** | .08 | **.13** | .17 | .00 | -.02 | -.04 | -.05 | -.11 | **-.12** | -.11 | -.03 | -.07 | .02 | -.01 | -.02 |
|  |  | zero-order | .09 | .12 | **.26** | **.30** | .19 | **.20** | .23 | -.04 | -.16 | -.11 | -.10 | -.22 | **-.18** | -.16 | -.14 | -.14 | -.04 | -.07 | -.05 |
| 125* | Became intoxicated. | partial | .01 | .05 | **.17** | **.21** | .09 | **.13** | .17 | -.02 | -.09 | -.10 | -.10 | **-.18** | **-.16** | **-.15** | -.05 | **-.14** | -.04 | -.07 | -.13 |
|  |  | zero-order | .04 | .08 | **.25** | **.29** | .18 | **.20** | .24 | -.08 | -.22 | -.16 | -.14 | **-.27** | **-.21** | **-.19** | -.16 | **-.22** | -.11 | -.14 | -.17 |
| 157* | Had a hangover. | partial | -.02 | .06 | **.17** | **.21** | .11 | **.14** | .17 | .04 | -.01 | -.07 | -.05 | -.08 | -.10 | -.10 | .01 | -.08 | -.02 | -.07 | -.10 |
|  |  | zero-order | .01 | .08 | **.23** | **.27** | .18 | **.22** | .23 | -.03 | -.15 | -.12 | -.09 | -.18 | -.16 | -.13 | -.10 | -.16 | -.11 | -.16 | -.16 |
| 187* | Drank alcohol during working hours. | partial | .01 | -.01 | .08 | .07 | -.01 | .03 | .06 | .01 | -.01 | .00 | -.09 | **-.14** | -.06 | -.06 | -.01 | -.03 | -.01 | -.05 | -.07 |
|  |  | zero-order | -.01 | -.03 | .04 | .01 | -.05 | .04 | .05 | -.01 | .00 | .02 | -.08 | **-.11** | -.06 | -.04 | .00 | -.04 | -.05 | -.09 | -.11 |
| *Physical activity* | | | | | | | | | | | | | | | | | | | | | |
| 6* | Went running or jogging. | partial | .13 | .10 | **.19** | .11 | .10 | .09 | .10 | .02 | -.03 | .06 | .14 | .07 | -.01 | .00 | .13 | .01 | .03 | .02 | .06 |
|  |  | zero-order | .18 | .15 | **.30** | .24 | .23 | .15 | .18 | -.02 | -.16 | -.04 | .08 | -.06 | -.08 | -.07 | -.01 | -.06 | .00 | -.01 | .05 |
| 100 | Played a team sport. | partial | -.05 | -.02 | .10 | -.01 | -.06 | .01 | -.10 | **-.10** | **-.14** | -.04 | .02 | -.04 | -.11 | .01 | -.01 | -.02 | -.05 | -.06 | -.01 |
|  |  | zero-order | .01 | .04 | .22 | .15 | .10 | .12 | .03 | **-.16** | **-.30** | -.12 | -.05 | -.19 | -.19 | -.07 | -.17 | -.14 | -.14 | -.15 | -.07 |
| 129 | Did aerobic exercise. | partial | .11 | .02 | .09 | .04 | .06 | .04 | .08 | .07 | .11 | .12 | .11 | .05 | .05 | .05 | **.14** | .09 | .09 | .03 | .04 |
|  |  | zero-order | .14 | .05 | .13 | .10 | .12 | -.01 | .06 | .10 | .11 | .07 | .11 | .05 | .06 | .02 | **.13** | .12 | .17 | .12 | .12 |
| *Self-development and learning* | | | | | | | | | | | | | | | | | | | | | |
| 7* | Attended a public lecture. | partial | **.28** | .08 | .15 | -.01 | .10 | .07 | .04 | .01 | -.01 | .12 | **.16** | .05 | .08 | .02 | .10 | .09 | .09 | .05 | .05 |
|  |  | zero-order | **.30** | .10 | .19 | .05 | .15 | .08 | .07 | .01 | -.05 | .08 | **.14** | .01 | .06 | -.01 | .06 | .07 | .09 | .06 | .06 |
| 36 | Looked up a word in a dictionary. | partial | **.24** | .07 | .08 | .00 | .07 | .05 | -.03 | .04 | -.01 | **.16** | .12 | .09 | .00 | -.02 | .07 | .09 | .09 | .06 | .07 |
|  |  | zero-order | **.27** | .10 | .15 | .09 | .15 | .07 | .01 | .03 | -.07 | **.10** | .09 | .02 | -.04 | -.06 | .00 | .05 | .09 | .05 | .08 |
| 30 | Watched an educational channel on TV. | partial | .13 | .00 | .06 | .05 | .03 | -.04 | .01 | .00 | .03 | .10 | .12 | .09 | .04 | .02 | **.16** | .05 | .07 | .05 | .08 |
|  |  | zero-order | .11 | -.01 | .02 | .01 | -.01 | -.05 | -.02 | .01 | .07 | .12 | .13 | .12 | .06 | .04 | **.18** | .07 | .07 | .05 | .08 |
| 40* | Visited an art exhibition. | partial | .19 | .02 | .02 | -.04 | -.03 | .00 | -.09 | -.09 | **-.15** | -.06 | -.08 | -.08 | -.08 | **-.12** | .05 | -.08 | .03 | -.03 | -.03 |
|  |  | zero-order | .20 | .03 | .04 | -.01 | .00 | .00 | -.08 | -.09 | **-.15** | -.07 | -.08 | -.09 | -.09 | **-.13** | .04 | -.08 | .05 | -.01 | -.01 |
| 72* | Visited a museum. | partial | **.21** | .06 | .03 | -.04 | -.02 | -.02 | -.09 | .03 | -.06 | .06 | .06 | .03 | .02 | -.04 | .13 | .05 | **.13** | .05 | .03 |
|  |  | zero-order | **.22** | .07 | .06 | -.01 | .01 | -.01 | -.08 | .03 | -.07 | .05 | .06 | .02 | .01 | -.05 | .11 | .04 | **.13** | .05 | .04 |
| 101* | Looked something up in an encyclopedia. | partial | .15 | .06 | .05 | -.04 | .04 | .00 | -.09 | .08 | -.02 | **.16** | .12 | .08 | .03 | .01 | .03 | .10 | .07 | .05 | .08 |
|  |  | zero-order | .17 | .08 | .10 | .02 | .10 | .02 | -.05 | .07 | -.07 | **.12** | .10 | .03 | .01 | -.02 | -.02 | .06 | .06 | .04 | .08 |
| 113* | Read a book. | partial | **.26** | .10 | .03 | -.03 | .01 | -.03 | -.09 | -.01 | -.02 | .04 | .05 | -.01 | -.06 | -.07 | .02 | .02 | .03 | .07 | .08 |
|  |  | zero-order | **.27** | .12 | .07 | .03 | .05 | -.05 | -.08 | .01 | -.02 | .02 | .05 | -.02 | -.06 | -.09 | .01 | .03 | .07 | .11 | .12 |
| 128* | Studied some subject. | partial | **.35** | **.20** | .10 | .06 | **.15** | .03 | .01 | **.12** | .06 | **.17** | .13 | .10 | .00 | .01 | .09 | **.15** | **.15** | **.17** | **.14** |
|  |  | zero-order | **.36** | **.21** | .14 | .11 | **.19** | .04 | .03 | **.11** | .02 | **.13** | .11 | .05 | -.02 | -.02 | .05 | **.13** | **.15** | **.17** | **.14** |
| 145* | Read poetry. | partial | .20 | .09 | .00 | -.08 | -.05 | -.05 | **-.18** | -.05 | **-.15** | .06 | .03 | .04 | -.03 | -.03 | **.17** | .07 | .10 | .06 | .04 |
|  |  | zero-order | .21 | .10 | .04 | -.03 | .00 | -.05 | **-.17** | -.04 | **-.14** | .03 | .02 | .02 | -.03 | -.05 | **.15** | .07 | .12 | .08 | .07 |
| 160* | Learned a new skill. | partial | **.27** | **.13** | .14 | .06 | **.14** | .05 | .02 | .05 | .01 | .06 | .02 | .03 | .02 | .00 | .05 | .08 | .09 | .09 | .04 |
|  |  | zero-order | **.30** | **.17** | .24 | .17 | **.24** | .10 | .08 | .02 | -.10 | -.01 | -.02 | -.07 | -.03 | -.06 | -.05 | .01 | .06 | .06 | .03 |
| 176* | Bought a book. | partial | **.27** | .07 | -.02 | -.08 | -.04 | -.03 | -.09 | -.05 | -.01 | .05 | .06 | .02 | -.06 | -.07 | .04 | .05 | **.13** | .04 | .07 |
|  |  | zero-order | **.27** | .07 | -.03 | -.08 | -.04 | -.06 | -.10 | -.03 | .03 | .05 | .06 | .04 | -.04 | -.06 | .06 | .07 | **.16** | .08 | .10 |
| 190 | Enrolled in a course of study. | partial | **.21** | .01 | .14 | .06 | .10 | .10 | .05 | .04 | .01 | .09 | .11 | .07 | .02 | .00 | .08 | .02 | .10 | .03 | .04 |
|  |  | zero-order | **.22** | .02 | .18 | .11 | .14 | .11 | .08 | .03 | -.03 | .05 | .09 | .02 | .00 | -.02 | .03 | .00 | .09 | .02 | .04 |
| *Creative hobby* | | | | | | | | | | | | | | | | | | | | | |
| 50* | Produced a work of art. | partial | .19 | .08 | .07 | -.01 | .07 | .04 | -.06 | -.02 | -.01 | .03 | -.02 | -.01 | -.04 | -.07 | **.13** | -.01 | .00 | .06 | .01 |
|  |  | zero-order | .20 | .10 | .10 | .04 | .10 | .05 | -.03 | -.02 | -.04 | .01 | -.03 | -.04 | -.06 | -.08 | **.10** | -.02 | .00 | .06 | .02 |
| 82* | Wrote poetry. | partial | .16 | .04 | .01 | -.06 | -.05 | -.02 | -.14 | -.09 | **-.14** | -.05 | -.06 | -.06 | -.08 | -.08 | .10 | -.01 | .00 | .06 | .01 |
|  |  | zero-order | .18 | .06 | .07 | .00 | .01 | .00 | -.10 | -.10 | **-.18** | -.08 | -.08 | -.10 | -.10 | -.11 | .05 | -.04 | .00 | .05 | .01 |
| 115 | Acted in a play. | partial | .02 | -.05 | .03 | -.07 | -.03 | .01 | -.06 | -.04 | **-.11** | -.02 | -.01 | -.11 | -.03 | -.06 | .02 | -.05 | -.05 | -.01 | -.04 |
|  |  | zero-order | .05 | -.03 | .08 | .00 | .03 | .02 | -.03 | -.04 | **-.14** | -.05 | -.03 | -.14 | -.05 | -.09 | -.02 | -.06 | -.04 | .00 | -.03 |
| 200* | Sang or played an instrument in public. | partial | .09 | -.02 | -.01 | -.08 | -.10 | -.02 | **-.19** | **-.12** | **-.17** | -.01 | .06 | .00 | -.05 | -.03 | -.01 | -.01 | .03 | .03 | .02 |
|  |  | zero-order | .12 | .02 | .06 | .00 | -.01 | .02 | **-.14** | **-.13** | **-.23** | -.06 | .04 | -.06 | -.08 | -.07 | -.07 | -.05 | .02 | .01 | .01 |
| 197* | Read a book about some artistic topic. | partial | **.24** | .06 | .12 | -.03 | .01 | .00 | -.08 | -.07 | -.07 | .01 | -.02 | -.04 | -.07 | -.10 | .10 | -.02 | .08 | .00 | -.01 |
|  |  | zero-order | **.24** | .06 | .12 | -.02 | .02 | -.01 | -.09 | -.06 | -.05 | .00 | -.01 | -.04 | -.06 | -.10 | .10 | -.01 | .09 | .02 | .01 |
| *Hobby* | | | | | | | | | | | | | | | | | | | | | |
| 89 | Traded something in my collection. | partial | .00 | -.07 | .04 | .01 | -.04 | .08 | -.02 | -.07 | -.08 | -.06 | -.04 | -.05 | -.09 | -.09 | .00 | -.08 | -.09 | -.09 | **-.13** |
|  |  | zero-order | .00 | -.08 | .03 | .00 | -.05 | .09 | -.02 | -.07 | -.08 | -.05 | -.04 | -.05 | -.09 | -.08 | .00 | -.08 | -.10 | -.11 | **-.15** |
| 127* | Read a fashion-related book. | partial | .04 | -.04 | **.17** | .11 | .11 | .07 | .13 | .05 | .09 | -.02 | .07 | .07 | **.13** | .02 | .09 | .03 | .06 | -.04 | -.03 |
|  |  | zero-order | .04 | -.04 | **.13** | .09 | .09 | .00 | .08 | .10 | .15 | -.01 | .09 | .11 | **.15** | .03 | .14 | .08 | .14 | .05 | .04 |
| *Playing games* | | | | | | | | | | | | | | | | | | | | | |
| 54* | Worked on a jigsaw puzzle. | partial | -.01 | -.04 | .00 | -.02 | -.08 | .00 | -.03 | .01 | .06 | .07 | .10 | **.13** | .05 | .04 | .06 | .05 | .02 | .00 | -.02 |
|  |  | zero-order | .01 | -.03 | .02 | .02 | -.04 | -.02 | -.03 | .02 | .06 | .05 | .10 | **.12** | .06 | .03 | .05 | .06 | .06 | .04 | .02 |
| 69* | Gambled with cards or dice. | partial | -.05 | -.09 | .06 | .04 | -.01 | .04 | .04 | -.04 | -.08 | -.07 | -.06 | **-.17** | -.04 | -.03 | -.04 | -.11 | -.04 | -.05 | -.11 |
|  |  | zero-order | -.05 | -.07 | .10 | .07 | .03 | .10 | .09 | -.08 | -.16 | -.09 | -.08 | **-.21** | -.08 | -.06 | -.10 | -.17 | -.11 | -.12 | -.16 |
| 119 | Played a board game. | partial | .03 | -.09 | -.06 | -.04 | -.10 | .01 | -.08 | **-.11** | -.01 | -.02 | .02 | -.05 | -.02 | -.04 | .01 | -.05 | -.02 | -.05 | .00 |
|  |  | zero-order | .06 | -.05 | .02 | .05 | -.01 | .04 | -.02 | **-.12** | -.09 | -.07 | -.01 | -.11 | -.05 | -.08 | -.06 | -.08 | -.03 | -.06 | .00 |
| 166* | Went to a casino. | partial | -.02 | -.04 | .09 | .10 | .02 | **.13** | .13 | .02 | .01 | -.05 | -.04 | -.05 | .03 | -.05 | .00 | -.03 | -.03 | -.08 | -.08 |
|  |  | zero-order | -.04 | -.05 | .08 | .07 | .00 | **.16** | .14 | -.02 | -.03 | -.04 | -.05 | -.06 | .01 | -.04 | -.02 | -.06 | -.10 | -.14 | -.13 |
| *Watching TV* | | | | | | | | | | | | | | | | | | | | | |
| 58 | Watched television news. | partial | .05 | .06 | .03 | .07 | .10 | -.03 | .05 | .08 | .08 | .11 | .12 | .10 | .04 | .02 | .06 | .01 | .05 | .10 | **.16** |
|  |  | zero-order | .02 | .03 | -.03 | .00 | .03 | -.04 | .02 | .08 | .12 | .14 | .14 | .14 | .06 | .05 | .10 | .03 | .05 | .10 | **.15** |
| 123 | Watched a television talk show. | partial | -.02 | .04 | .09 | .13 | .13 | .03 | **.18** | .08 | .08 | .01 | .02 | .05 | .03 | -.01 | .01 | -.02 | -.01 | .05 | .04 |
|  |  | zero-order | .00 | .06 | .12 | .17 | .17 | .03 | **.19** | .08 | .05 | -.02 | .01 | .02 | .01 | -.03 | -.01 | -.03 | .00 | .06 | .06 |
| 155 | Watched a television reality show. | partial | -.08 | -.01 | .09 | .15 | .13 | .09 | .15 | **.12** | .09 | .00 | .05 | .03 | .06 | .00 | .04 | .00 | -.04 | .03 | .02 |
|  |  | zero-order | -.06 | .01 | .12 | .18 | .16 | .09 | .16 | **.12** | .06 | -.03 | .05 | .00 | .05 | -.02 | .02 | -.01 | -.03 | .04 | .03 |
| *Music* | | | | | | | | | | | | | | | | | | | | | |
| 207* | Used an mp3 player or Ipod. | partial | .18 | **.14** | .15 | .12 | .08 | .08 | .03 | -.01 | -.09 | -.04 | -.04 | -.05 | -.11 | **-.17** | -.05 | -.04 | .11 | .00 | .03 |
|  |  | zero-order | .22 | **.19** | .29 | .29 | .24 | .15 | .13 | -.06 | -.25 | -.15 | -.10 | -.20 | -.18 | **-.23** | -.19 | -.14 | .04 | -.05 | .01 |
| *Using Internet (besides communication)* | | | | | | | | | | | | | | | | | | | | | |
| 65* | Downloaded music from the Internet. | partial | .08 | .05 | **.18** | .13 | **.14** | .05 | .09 | .01 | -.09 | -.07 | -.04 | -.09 | -.06 | -.06 | -.09 | -.03 | .04 | .04 | .03 |
|  |  | zero-order | .15 | .13 | **.35** | .34 | **.33** | .16 | .21 | -.06 | -.30 | -.20 | -.11 | -.27 | -.16 | -.16 | -.26 | -.16 | -.03 | -.04 | .00 |
| 206* | Used eBay to buy or sell something. | partial | .12 | .09 | .07 | .04 | **.14** | .01 | .09 | .01 | -.04 | -.01 | -.01 | -.03 | -.11 | -.10 | .00 | -.03 | .04 | .01 | .03 |
|  |  | zero-order | .15 | .12 | .15 | .13 | **.21** | .07 | .15 | -.02 | -.13 | -.06 | -.04 | -.11 | -.16 | -.14 | -.08 | -.08 | .00 | -.03 | .02 |
| *Travelling* | | | | | | | | | | | | | | | | | | | | | |
| 44 | Went on a hike. | partial | .09 | -.02 | .02 | -.04 | -.10 | -.05 | -.11 | -.07 | **-.13** | -.05 | -.04 | -.06 | -.08 | -.05 | .07 | -.06 | .02 | -.04 | -.05 |
|  |  | zero-order | .11 | .00 | .06 | .02 | -.05 | -.03 | -.08 | -.08 | **-.17** | -.08 | -.05 | -.10 | -.10 | -.07 | .03 | -.08 | .02 | -.05 | -.05 |
| 170 | Went on a cruise or tour. | partial | .09 | -.01 | .14 | .03 | .00 | .01 | -.04 | .00 | -.02 | .06 | .11 | .02 | **.12** | .05 | .07 | .10 | .06 | .00 | .07 |
|  |  | zero-order | .11 | .01 | .18 | .08 | .05 | .03 | -.01 | -.01 | -.07 | .02 | .09 | -.02 | **.09** | .02 | .02 | .07 | .05 | .00 | .07 |
| *Social interaction* | | | | | | | | | | | | | | | | | | | | | |
| 34* | Sent a message by electronic mail (e-mail). | partial | .16 | **.12** | .00 | -.01 | .06 | -.04 | .00 | .02 | .03 | .06 | .04 | .07 | -.05 | -.06 | .03 | .05 | .10 | .07 | .09 |
|  |  | zero-order | .21 | **.16** | .12 | .12 | .18 | .00 | .06 | .00 | -.08 | -.03 | .00 | -.03 | -.10 | -.11 | -.07 | -.01 | .09 | .06 | .10 |
| 97* | Traded music with a friend. | partial | .04 | .02 | .16 | .14 | .07 | .05 | .04 | -.07 | **-.14** | -.11 | -.07 | -.12 | -.05 | **-.13** | -.04 | -.05 | .07 | -.02 | .00 |
|  |  | zero-order | .10 | .09 | .30 | .30 | .23 | .12 | .13 | -.09 | **-.27** | -.20 | -.11 | -.24 | -.12 | **-.20** | -.17 | -.13 | .04 | -.04 | .01 |
| 108* | Participated in an online discussion group. | partial | .14 | .02 | **.17** | .09 | .08 | .10 | .05 | .00 | -.05 | .01 | .01 | -.04 | -.05 | -.11 | -.01 | .00 | **.12** | .02 | .03 |
|  |  | zero-order | .18 | .08 | **.28** | .23 | .21 | .17 | .13 | -.05 | -.20 | -.08 | -.05 | -.17 | -.12 | -.17 | -.14 | -.09 | **.06** | -.04 | .01 |
| *Dating and partying* | | | | | | | | | | | | | | | | | | | | | |
| 85 | Went dancing. | partial | .08 | .05 | .13 | **.18** | .09 | .06 | .10 | .04 | .04 | .06 | .08 | .03 | .04 | -.01 | .05 | .02 | .03 | .05 | .05 |
|  |  | zero-order | .13 | .11 | .24 | **.30** | .21 | .08 | .15 | .03 | -.05 | -.03 | .04 | -.07 | -.01 | -.07 | -.04 | -.03 | .05 | .07 | .08 |
| 78* | Went to a large party. | partial | .06 | **.12** | **.19** | **.20** | **.14** | **.12** | **.18** | .03 | -.02 | -.01 | .03 | -.09 | -.07 | -.08 | -.03 | -.03 | .05 | .03 | .05 |
|  |  | zero-order | .11 | **.16** | **.29** | **.31** | **.25** | **.16** | **.24** | .00 | -.13 | -.09 | -.02 | -.18 | -.12 | -.14 | -.12 | -.09 | .03 | .01 | .05 |
| 143 | Entertained six or more people. | partial | .02 | .04 | .16 | **.17** | .10 | .08 | .03 | .04 | -.04 | -.01 | .07 | -.03 | .02 | -.04 | .03 | -.01 | .08 | .06 | .05 |
|  |  | zero-order | .03 | .03 | .14 | **.15** | .08 | .06 | .02 | .05 | -.01 | .00 | .08 | -.01 | .03 | -.03 | .05 | .01 | .10 | .08 | .07 |
| 172 | Used an online dating web-side. | partial | -.05 | -.08 | .02 | .01 | -.08 | .04 | .02 | -.04 | -.02 | -.11 | -.04 | -.02 | .01 | -.03 | -.02 | .01 | -.02 | **-.11** | -.09 |
|  |  | zero-order | -.03 | -.06 | .06 | .06 | -.03 | .06 | .05 | -.05 | -.06 | -.13 | -.05 | -.05 | -.01 | -.06 | -.06 | -.02 | -.02 | **-.11** | -.08 |
| *Transportation* | | | | | | | | | | | | | | | | | | | | | |
| 59* | Rode a motorcycle. | partial | -.03 | .01 | .13 | .06 | .08 | **.13** | .12 | .05 | -.01 | -.02 | .03 | .01 | -.01 | -.02 | .04 | -.02 | -.04 | .01 | -.06 |
|  |  | zero-order | -.02 | .02 | .17 | .10 | .12 | **.18** | .16 | .01 | -.10 | -.05 | .00 | -.05 | -.05 | -.04 | -.03 | -.08 | -.10 | -.06 | -.10 |
| 156 | Raced a car, truck or motorcycle. | partial | -.02 | .04 | **.17** | .14 | .08 | .10 | .14 | .06 | .04 | .02 | .08 | .04 | .00 | -.02 | .06 | -.03 | -.01 | .04 | -.02 |
|  |  | zero-order | -.03 | .04 | **.17** | .14 | .09 | .16 | .17 | .01 | -.05 | .01 | .04 | -.02 | -.04 | -.03 | -.01 | -.09 | -.10 | -.05 | -.09 |
| *Religious practices* | | | | | | | | | | | | | | | | | | | | | |
| 2* | Discussed religion or spirituality. | partial | .13 | .02 | -.08 | -.11 | -.11 | -.09 | -.13 | -.07 | .02 | **.16** | **.18** | .10 | .07 | .05 | .11 | .12 | .05 | .08 | .10 |
|  |  | zero-order | .12 | .01 | -.10 | -.12 | -.12 | -.09 | -.14 | -.07 | .04 | **.17** | **.19** | .11 | .08 | .06 | .12 | .13 | .06 | .08 | .10 |
| 33* | Prayed (not including blessings at meals). | partial | .03 | -.04 | -.07 | -.12 | .00 | -.04 | -.07 | .01 | .11 | **.17** | **.33** | **.17** | **.17** | **.14** | .07 | **.17** | .05 | .10 | .11 |
|  |  | zero-order | .01 | -.06 | -.13 | -.18 | -.07 | -.09 | -.12 | .05 | .19 | **.21** | **.35** | **.23** | **.20** | **.17** | .14 | **.22** | .09 | .14 | .13 |
| 66* | Read the Bible or other sacred text. | partial | .13 | .01 | -.10 | **-.17** | -.09 | -.07 | **-.22** | -.03 | -.01 | **.17** | **.30** | **.12** | **.13** | .10 | .10 | **.18** | .06 | **.13** | **.13** |
|  |  | zero-order | .12 | .01 | -.10 | **-.17** | -.09 | -.08 | **-.23** | -.02 | .01 | **.17** | **.30** | **.13** | **.13** | .11 | .11 | **.19** | .07 | **.14** | **.14** |
| 98* | Gave a blessing at a meal. | partial | .08 | -.10 | -.08 | -.13 | -.09 | -.06 | **-.18** | -.06 | -.02 | **.15** | **.33** | **.15** | **.19** | **.14** | .07 | **.18** | .03 | .11 | .12 |
|  |  | zero-order | .06 | -.11 | -.12 | -.17 | -.13 | -.08 | **-.21** | -.05 | .03 | **.18** | **.34** | **.18** | **.21** | **.16** | .10 | **.20** | .04 | .11 | .12 |
| 131* | Attended a church or religious service. | partial | .03 | -.04 | -.06 | -.12 | -.01 | -.04 | -.10 | .02 | .05 | **.17** | **.32** | **.14** | **.12** | **.15** | .01 | **.15** | .03 | **.12** | **.13** |
|  |  | zero-order | .01 | -.05 | -.10 | -.15 | -.05 | -.08 | -.14 | .05 | .11 | **.19** | **.33** | **.18** | **.15** | **.17** | .06 | **.19** | .07 | **.15** | **.15** |
| 163* | Listened to a religious program on the radio or TV. | partial | .09 | -.10 | -.08 | **-.18** | -.13 | -.01 | **-.18** | -.06 | -.03 | **.18** | **.33** | .12 | **.17** | **.18** | .10 | .14 | .04 | .03 | .06 |
|  |  | zero-order | .05 | -.14 | -.17 | **-.27** | -.22 | -.06 | **-.23** | -.02 | .08 | **.23** | **.35** | .20 | **.21** | **.22** | .18 | .19 | .07 | .06 | .07 |
| 193* | Read a book about religion or spirituality. | partial | .18 | .05 | -.07 | **-.17** | -.08 | .01 | **-.18** | -.09 | -.02 | .14 | **.25** | .07 | .08 | .07 | .10 | .12 | .06 | .04 | .08 |
|  |  | zero-order | .16 | .03 | -.12 | **-.22** | -.13 | -.02 | **-.21** | -.07 | .05 | .17 | **.27** | .12 | .11 | .09 | .14 | .15 | .08 | .07 | .09 |
| *Environmental activities* | | | | | | | | | | | | | | | | | | | | | |
| 55* | Composted food scraps or yard waste. | partial | .10 | -.01 | -.04 | -.09 | -.10 | -.09 | -.15 | -.04 | .00 | .05 | .07 | .10 | .06 | .07 | **.20** | .02 | .11 | .08 | .04 |
|  |  | zero-order | .05 | -.04 | -.13 | -.18 | -.18 | -.12 | -.19 | -.02 | .08 | .10 | .09 | .17 | .09 | .11 | **.25** | .06 | .11 | .08 | .03 |
| 120* | Changed a habit to have less impact on the environment. | partial | .12 | .00 | .07 | .01 | .02 | .01 | -.03 | .00 | **.12** | .13 | .12 | **.12** | .04 | .06 | **.28** | .11 | .11 | .05 | .05 |
|  |  | zero-order | .11 | -.01 | .02 | -.03 | -.03 | -.02 | -.06 | .02 | **.17** | .15 | .14 | **.16** | .07 | .08 | **.31** | .14 | .13 | .08 | .06 |
| 152* | Used both sides of a piece of paper before discarding it. | partial | .15 | .04 | -.04 | -.09 | .01 | -.07 | -.13 | .05 | .05 | .14 | .09 | .09 | .02 | .05 | .13 | .10 | .11 | **.13** | .08 |
|  |  | zero-order | .17 | .06 | .00 | -.04 | .06 | -.06 | -.10 | .04 | .01 | .11 | .08 | .05 | .01 | .02 | .09 | .08 | .11 | **.12** | .09 |
| 192* | Picked up litter. | partial | .10 | .01 | .03 | -.02 | -.03 | -.05 | -.14 | -.02 | .01 | .09 | .10 | .09 | .07 | .06 | .13 | **.14** | **.14** | .10 | .07 |
|  |  | zero-order | .08 | -.01 | -.02 | -.07 | -.08 | -.07 | -.17 | -.01 | .07 | .12 | .11 | .13 | .10 | .08 | .16 | **.17** | **.15** | .11 | .07 |
| *Finanse/Investment* | | | | | | | | | | | | | | | | | | | | | |
| 16* | Obtained stock market prices. | partial | .11 | .02 | .14 | .07 | .04 | **.16** | .09 | .05 | .06 | -.01 | -.02 | .01 | -.03 | .04 | .03 | -.01 | .03 | -.03 | -.02 |
|  |  | zero-order | .09 | .01 | .11 | .03 | .01 | **.17** | .09 | .03 | .05 | .01 | -.02 | .02 | -.03 | .05 | .03 | -.02 | -.01 | -.07 | -.06 |
| 56* | Read a book on a financial topic. | partial | .16 | .06 | .14 | .03 | .10 | **.13** | .10 | .03 | .06 | .02 | .08 | .10 | .00 | .02 | .08 | .00 | .08 | .00 | -.02 |
|  |  | zero-order | .13 | .04 | .09 | -.02 | .05 | **.12** | .08 | .03 | .08 | .05 | .08 | .12 | .01 | .04 | .10 | .01 | .06 | -.01 | -.04 |
| 116* | Donated money to a political campaign or cause. | partial | .00 | **-.16** | .07 | -.05 | -.04 | .10 | .07 | .01 | -.01 | -.05 | .02 | -.05 | -.01 | -.01 | .03 | -.03 | -.05 | -.10 | **-.14** |
|  |  | zero-order | -.02 | **-.17** | .03 | -.09 | -.07 | .10 | .05 | .01 | .01 | -.03 | .02 | -.02 | .00 | .01 | .05 | -.02 | -.06 | -.11 | **-.15** |
| *Others* | | | | | | | | | | | | | | | | | | | | | |
| 17* | Tried something completely new. | partial | .17 | .09 | **.18** | .12 | .09 | .08 | .02 | .04 | -.03 | .00 | -.06 | -.02 | -.06 | -.04 | .06 | -.02 | .05 | .02 | .00 |
|  |  | zero-order | .20 | .13 | **.27** | .22 | .19 | .13 | .09 | .01 | -.14 | -.07 | -.10 | -.12 | -.11 | -.09 | -.04 | -.08 | .02 | -.01 | -.01 |
| 67 | Spent more than an hour thinking about what to wear. | partial | .06 | .01 | .15 | .15 | .10 | .10 | .15 | **.11** | .04 | .00 | .05 | .01 | .08 | -.02 | .08 | .06 | .03 | .03 | .01 |
|  |  | zero-order | .09 | .03 | .16 | .17 | .13 | .03 | .11 | **.15** | .09 | -.02 | .06 | .03 | .10 | -.03 | .09 | .10 | .13 | .13 | .10 |
| 96 | Ate in a restaurant. | partial | **.21** | **.10** | .12 | .13 | .10 | .03 | .05 | .04 | .00 | .05 | .07 | .03 | -.02 | -.05 | .03 | .02 | .09 | .09 | .07 |
|  |  | zero-order | **.23** | **.13** | .17 | .18 | .15 | .06 | .09 | .03 | -.06 | .01 | .05 | -.03 | -.05 | -.08 | -.02 | -.01 | .07 | .07 | .07 |
| 174* | Worked on a scrap book. | partial | .08 | .00 | .04 | .03 | -.03 | .01 | -.04 | .03 | .00 | -.01 | **.17** | .05 | .06 | -.01 | .00 | .01 | .06 | .05 | .09 |
|  |  | zero-order | .09 | .01 | .04 | .04 | -.01 | -.03 | -.05 | .06 | .03 | -.01 | **.17** | .06 | .07 | -.02 | .02 | .04 | .10 | .10 | .13 |
| 180* | Participated in an animal show. | partial | .02 | -.04 | .08 | .02 | .01 | .06 | .02 | -.01 | .04 | .03 | .05 | .01 | .04 | .05 | **.14** | .02 | .02 | -.02 | -.02 |
|  |  | zero-order | .00 | -.06 | .02 | -.04 | -.04 | .03 | -.01 | .00 | .09 | .06 | .07 | .06 | .06 | .07 | **.18** | .04 | .02 | -.01 | -.02 |
| 181 | Wore formal clothing. | partial | .09 | .03 | .05 | .02 | .09 | .05 | .06 | .09 | .09 | .13 | .15 | .09 | .06 | .07 | .12 | .11 | .12 | **.14** | .09 |
|  |  | zero-order | .10 | .03 | .05 | .03 | .09 | .03 | .05 | .11 | .10 | .13 | .15 | .10 | .06 | .07 | .13 | .13 | .14 | **.16** | .12 |
| 188* | Attended a town meeting. | partial | .03 | -.06 | .03 | .01 | .01 | **.14** | .04 | -.03 | .00 | .00 | .04 | -.01 | -.01 | -.02 | .02 | -.01 | .03 | -.05 | -.06 |
|  |  | zero-order | -.01 | -.09 | -.05 | -.08 | -.07 | **.12** | .00 | -.03 | .05 | .05 | .06 | .04 | .02 | .02 | .07 | .01 | .01 | -.06 | -.08 |

*Note.* SDT = self-direction-thought; SDA = self-direction-action; ST = stimulation; HE = hedonism; AC = achievement; POD = power-dominance; POR = power-resources; FAC = face; SEP = security-personal; SES = security-societal; TR = tradition; COR = conformity-rules; COI = conformity-interpersonal; HU = humility; UNN = universalism-nature; UNC = universalism-concern; UNT = universalism-tolerance; BED = benevolence-dependability; BEC = benevolence-caring.

*N* = 703. All coefficients > |.07| are significant at *p* < .05. All coefficients > |.10| are significant at *p* < .01. All coefficients > |.12| are significant at *p* < .001.

*Behavioral item included also in the highest correlates of centered value scores (Table A).

The 10 highest correlates for each value are in bold.
